# Enhancement of cytokine‐driven NK cell IFN‐γ production after vaccination of HCMV infected Africans

**DOI:** 10.1002/eji.201746974

**Published:** 2017-04-24

**Authors:** Alansana Darboe, Ebrima Danso, Ed Clarke, Ama Umesi, Ebrima Touray, Rita Wegmuller, Sophie E. Moore, Eleanor M. Riley, Martin R. Goodier

**Affiliations:** ^1^ Department of Immunology and Infection London School of Hygiene and Tropical Medicine London UK; ^2^ MRC International Nutrition Group Nutrition Theme MRC Keneba Cambridge UK; ^3^ Vaccine and Immunity Theme Infant Immunology Medical Research Council Unit The Gambia Cambridge UK; ^4^ MRC Human Nutrition Research Cambridge UK

**Keywords:** DTPiP vaccine, Influenza vaccine, NK cells, NKG2C, Vaccination

## Abstract

Human cytomegalovirus (HCMV) infection drives the phenotypic and functional differentiation of NK cells, thereby influencing the responses of these cells after vaccination. NK cell functional differentiation is particularly advanced in African populations with universal exposure to HCMV. To investigate the impact of advanced differentiation on vaccine‐induced responses, we studied NK‐cell function before and after vaccination with Trivalent Influenza Vaccine (TIV) or diphtheria, tetanus, pertussis, inactivated poliovirus vaccine (DTPiP) in Africans with universal, lifelong HCMV exposure. In contrast to populations with lower prevalence of HCMV infection, no significant enhancement of NK‐cell responses (IFN‐γ, CD107a, CD25) occurred after in vitro re‐stimulation of post‐vaccination NK cells with TIV or DTPiP antigens compared to pre‐vaccination baseline cells. However, both vaccinations resulted in higher frequencies of NK cells producing IFN‐γ in response to exogenous IL‐12 with IL‐18, which persisted for up to 6 months. Enhanced cytokine responsiveness was restricted to less differentiated NK cells, with increased frequencies of IFN‐γ^+^ cells observed within CD56^bright^CD57^−^, CD56^dim^CD57^−^NKG2C^−^ and CD56^dim^CD57^−^NKG2C^+^ NK‐cell subsets. These data suggest a common mechanism whereby different vaccines enhance NK cell IFN‐γ function in HCMV infected donors and raise the potential for further exploitation of NK cell “pre‐activation” to improve vaccine effectiveness.

## Introduction

Natural killer (NK) cells are classically regarded as mediators of innate immune responses, lysing infected or otherwise diseased cells and secreting cytokines within hours to days of initiation of an immune response 1. However, NK cells can also contribute as effector cells to adaptive immune responses by mediating antigen‐specific antibody‐dependent cellular cytotoxicity (ADCC) 2, 3 and producing IFN‐γ in response to interleukin‐2 (IL‐2) secreted by antigen‐specific CD4^+^ T cells 2, 4, 5, 6, 7. Moreover, it is recognized that NK cells undergo intrinsic transformation as a result of infection or inflammation 3, 8, 9, 10, 11.

Recent evidence increasingly suggests that NK cells can display certain characteristics of immunological memory. However, this phenomenon has been described in different models. First, murine cytomegalovirus‐ and hapten‐induced memory NK cells were described in human and mice 12, 13. Second, cytokine‐induced memory‐like NK cells generated through the pre‐activation of NK cells with interleukin (IL)‐12/18/15 cytokines 14, 15. Finally, “adaptive” NK cells mainly identified in HCMV infected individuals with increased expression of CD57, leukocyte immunoglobulin‐like receptor, and CD2 with or without NKG2C as well as decreased CD161 and CD7 and lack of the signaling adaptor FcɛRγI‐, and transcription factors such as Syk, EAT‐2 and promyelocytic leukemia zinc finger (PLZF) 11, 16, 17, 18. All of these scenarios demonstrate characteristics of enhanced NK cell functionality post‐infection and/or stimulation.

We, and others, have shown that NK cells make a significant contribution to vaccine‐induced immune responses to intracellular pathogens, including viruses. In vitro stimulation of peripheral blood mononuclear cells (PBMC) from vaccinated individuals with vaccine antigens including rabies 5, malaria 19, tetanus toxoid 20, whole cell *Bordetella pertussis* 6, and inactivated influenza virus 2, 6, results in NK cell degranulation (surface expression of CD107a), IFN‐γ production and upregulation of IL‐2Rα/ CD25. NK cell IFN‐γ responses to vaccine antigens are enhanced after vaccination with trivalent influenza vaccines, rabies or therapeutic HIV, in a T cell IL‐2 dependent process 2, 5, 7. Moreover, antibodies contribute to NK cell CD107a and CD25 expression through formation of immune complexes and cross‐linking of FcγRIII (CD16) and these responses may also be enhanced by vaccination 2, 21, 22, 23.

Different subpopulations (subsets) of NK cells contribute differentially to vaccine driven responses. CD56^bright^ and CD57^−^ NK cells are primarily activated to produce IFN‐γ by exogenous cytokines (including IL‐2, IL‐12, IL‐15, IL‐18, and IL‐21 20, 24, 25, 26. CD56^dim^CD57^+^ NK cells respond less effectively to cytokines (due, in part, to reduced expression of cell surface cytokine receptors) 9 but secrete IFN‐γ and become cytotoxic in response to ligation of activating surface receptors such as natural cytotoxicity receptors (NCRs) and CD16 20, 25, 26. Furthermore, the proportions of NK cells in each of these subsets varies between individuals according to age, genotype and prior exposure to infections 25, 26, 27, 28.

In particular, human cytomegalovirus (HCMV) is a known modulator of NK cell phenotype and function, predominantly associated with ligation of the activating C‐type lectin‐like receptor, CD94/NKG2C, by an HCMV peptide (UL40) bound to HLA‐E, but also with expansions of NK cells expressing activating Killer cell Immunoglobulin‐like Receptors (KIR) 10, 29, 30, 31. HCMV‐seropositive individuals accumulate “adaptive” NK cells, which are mostly CD56^dim^, CD57^+^ and CD16^bright^, and frequently (but not always) express NKG2C 3, 11, 29. In keeping with this phenotype, adaptively expanded NK cells are less responsive to exogenous IL‐12 and IL‐18 but are highly efficient mediators of ADCC (including killing of HCMV infected target cells) and produce IFN‐γ in response to cross‐linking of CD16 9, 11.

We have observed that NK cells from HCMV‐seropositive subjects make low frequency IFN‐responses to killed or inactivated pathogens or vaccine antigens and that responses are not strongly enhanced by vaccination 2, 6, 20. However, these impacts of HCMV‐infection are not only due to skewing of NK cell populations towards an “adaptive” phenotype as IFN‐γ responses are lower in all NK subsets of HCMV seropositive individuals when compared to HCMV seronegative individuals 6.

The seroprevalence of HCMV increases with age and varies enormously between populations 32, 33. HCMV serostatus is therefore likely to be a significant confounder of comparisons of immune responsiveness over the life course and between populations. We have previously studied the phenotype and function of NK cells in a Gambian population (age range 1 and 49 years) where HCMV infection is essentially universal by the age of 12 months (>97% HCMV seropositive) 28. In this population, differentiation of NK cells from CD56^bright^ through CD56^dim^CD57^−^ to CD56^dim^CD57^+^ occurs very early in life and is maximal by the age of 10 years; acquisition of CD57 is often associated with expression of NKG2C and NK cell differentiation is delayed in individuals lacking NKG2C due to a homozygous deletion of *KLRC2*, the locus encoding NKG2C 28.

We therefore investigated whether NK cells from Gambian children would produce IFN‐γ in response to pathogen‐associated vaccines and whether vaccination would significantly boost these responses. Moreover, we have recently observed that vaccination can enhance NK cell IFN‐γ responses to exogenous cytokines (in a manner analogous to in vitro “pre‐activation” of NK cells by IL‐12 and IL‐18 14, 15, 34 in HCMV‐seropositive but not HCMV‐seronegative donors 2. We therefore hypothesized that vaccination would enhance NK‐cell responsiveness to cytokines in this HCMV‐infected population.

We investigated the impact of two different vaccinations on NK cell function and phenotype in The Gambia. First, we studied the NK cell response to primary vaccination with inactivated, trivalent, seasonal influenza vaccine (TIV) in a previously unvaccinated, age‐stratified cohort. Second, the NK cell response to booster vaccination against diphtheria, tetanus, pertussis, and polio (DTPiP) was examined in adults who had been vaccinated in infancy. We observed only limited enhancement of TIV‐driven T‐cell‐dependent NK‐cell responses after vaccination in all age groups. However, significant enhancement of vaccine antigen independent responses to exogenous pro‐inflammatory cytokines was observed, persisting for at least 6 months after vaccination.

## Results

### High HCMV seroprevalence in Gambian children and adults

For the TIV study, complete sample sets (baseline, 1, 3, and 6 months post‐vaccination) were obtained from 68 individuals; 22 children (2‐6 years of age), 21 young adults (20‐30 years or age) and 25 older adults (60‐75 years of age) (Table 1). Similar numbers of male and female subjects were recruited within each age group (Table 1). As expected, HCMV was highly prevalent with only one child and one elderly adult being seronegative (Table 1). Median anti‐HCMV IgG concentrations were slightly, but not significantly, higher in children than 20–30 year young adults and were significantly higher among 60–75 year olds compared to young adults, indicating potential HCMV reactivation in the oldest individuals. The vast majority of subjects were also seropositive for EBV infection with a tendency toward increasing seroprevalence with increasing age but without any significant age‐dependent effect on EBNA‐1 IgG titres (Table 1). Additionally, the overall frequencies of individuals homozygous for the NKG2C wild type gene was 44.1%; 42.6% were heterozygous and 13.2% were homozygous for the *NKG2C* gene deletion (an allele frequency of 34.6%) consistent with known frequency in The Gambia 35 (Table 1).

**Table 1:** Cohort characteristics: Baseline NKG2C genotype, HCMV and EBV IgG antibody levels

Age group (years)	n(male/female)	Median age	HCMV IgG+ n (%)	HCMV IgG titer, IU/mL median (range)	EBV nuclear antigen IgG+, n (%)	EBV nuclear antigen IgG titer IU/mL, median (range)	NKG2C genotype n (%)		
							+/+	+/−	−/−
2‐6	22 (11/11)	3.9	21 (95.5)	398 (35‐2942)	19 (86.4)	76 (17‐185)	12 (54.5)	7 (31.8)	3 (13.6)
20‐30	21 (13/8)	21.7	21 (100)	238 (54‐798)∞	19 (90.5)	103 (8‐178)	7 (33.3)	10 (47.6)	4 (19.0)
60‐75	25 (13/12)	65.0	24 (96.0)	580 (72‐8618)∞	24 (96.0)	76 (3‐192)	11(44.0)	12 (48.0)	2 (8.0)
Total	68 (37/31)		66 (97.1)	398 (35‐8618)	62 (91.2)	85 (3‐185)	30 (44.1)	29 (42.6)	9 (13.2)

^∞^HCMV IgG antibody levels significantly different between the young adults and oldest age groups (*p < 0.05).

### Age‐related changes in NK‐cell differentiation phenotype

**Figure 1:**
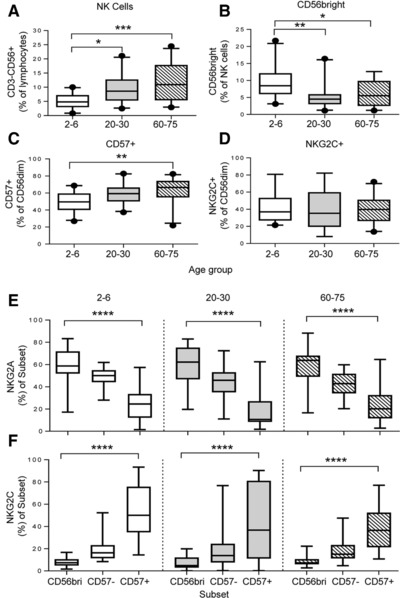
Age‐dependent differences in NK‐cell subsets. (A–F) Proportions of NK cells and subsets were determined ex‐vivo at baseline for three age‐defined groups (2–6, 20–30, 60–75 years). Proportions of (A) CD56^+^CD3^−^ NK cells within total lymphocytes and (B) CD56^bright^ cells within NK cells. Frequency of (C) CD57 and (D) NKG2C^+^ cells within CD56^dim^ NK cells. Expression of (E) NKG2A and (F) NKG2C within CD56/CD57‐defined NK cell subsets. Data are shown for 68 subjects. Boxes indicate median values with interquartile ranges and whiskers indicate 95th percentiles. Statistical analysis was performed on samples using (A–D) Kruskal–Wallis test, *p < 0.05, **p < 0.01, ***p < 0.001 and (E,F) using linear trend ANOVA with correction for multiple comparisons ****p < 0.0001.

Both HCMV infection and age influence the differentiation and function of NK cells and may therefore affect vaccine responses 25, 26, 28. PBMC collected at baseline (prior to vaccination) from participants in the influenza study were therefore analysed ex vivo for NK cell (Fig. 1; flow cytometry gating strategies are shown for NK cells in Supporting Information Fig. 1, blood myeloid and lymphoid lineages in Supporting Information Fig. 2 and memory T cells in Supporting Information Fig. 3).

The overall frequency of NK cells (CD3^−^CD56^+^) among the peripheral lymphocyte population increased significantly with increasing age (Fig. 1A) and, within the NK cell population, the frequency of CD56^bright^ NK cells was significantly higher among children than among adults (Fig. 1B). While there was a gradated increase in the frequencies of cells expressing the late differentiation marker CD57 (Fig. 1C), similar frequencies of NKG2C^+^ NK cells were observed at all ages (Fig. 1D). As expected, the frequency of cells expressing NKG2A decreased, and the frequency of cells expressing NKG2C increased, as NK cells differentiated from CD56^bright^ via CD56^dim^CD57^−^ to CD56^dim^CD57^+^ (Fig. 1E and F). No significant difference was observed in the frequency of highly differentiated CD57^+^NKG2C^+^ NK cells between children and adults in this cohort (Fig. 1E and F).

B‐cell frequencies were significantly higher in 2–6 year‐old children than in adults and there was a tendency for the frequencies of blood myeloid cell populations to increase with age (Supporting Information Fig. 2). While the overall proportion of CD3^+^ T cells did not differ between age groups, both CD4^+^ and CD8^+^ T cells differentiated toward effector memory cell populations with increasing age (Supporting Information Fig. 3). There was a particularly marked accumulation of highly differentiated CD28^−^CD57^+^CD4^+^ T cells in the oldest age group (Supporting Information Fig. 3, E), consistent with previous observations in the elderly 36, 37. While the proportions of CD28^−^CD57^+^ CD8^+^ T cells that were highest in the oldest age group, high frequencies were also present in children, as observed previously by ourselves and others (Supporting Information Fig. 3, J) 37, 38.

### Effect of vaccination on NK‐cell responses to influenza vaccine antigens

We have previously observed, in UK subjects, that natural exposure to influenza, or vaccination with TIV, promotes T‐cell‐dependent NK‐cell IFN‐γ responses and antibody dependent NK cell degranulation 2, 6. Importantly, upregulation of CD25 and production of IFN‐γ by NK cells after in vitro restimulation with vaccine antigens was consistently greater among HCMV seronegative than HCMV seropositive subjects, whereas degranulation responses were relatively unaffected by HCMV infection 2, 6. Thus, given the very high prevalence of HCMV infection in The Gambia (Table 1), we hypothesized that vaccination of Gambian subjects with TIV might potentiate antigen/antibody‐induced degranulation responses but not IFN‐γ production. A potential exception to such a response pattern could be in 2–6 year‐old children, among whom the effects of persistent HCMV infection may not yet be fully apparent.

**Figure 2:**
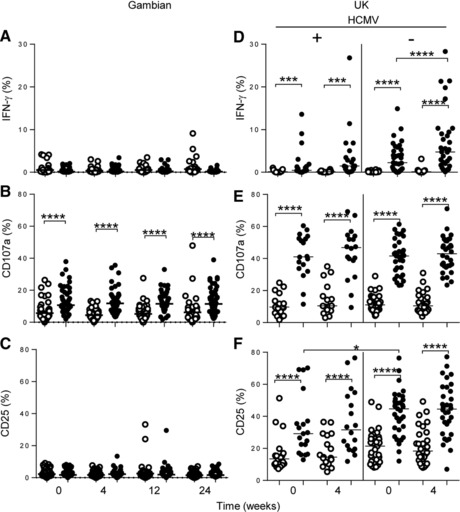
No change in NK cell responses to TIV after vaccination. (A–F) Frequencies of IFN‐γ (A, D), CD107a^+^ (B, E) and CD25^+^ (C, F) NK cells were determined after in vitro stimulation of PBMC from (A–C) TIV vaccinated Gambians at baseline and 4, 12, and 24 weeks after vaccination. (D–F) Data from HCMV seropositive and seronegative UK subjects at baseline and 4 weeks post TIV vaccination. Cells were cultured in (LCC) low concentration of cytokines (rIL‐12: & rIL18) alone (open symbols) or LCC plus TIV vaccine antigen (closed symbols) (A–F). Data are shown for (A–C) 61 TIV vaccinated Gambian and (D–F) 52 UK subjects, comprising 19 HCMV+ and 33 HCMV‐ individuals. Dots represent individual values and horizontal lines represent the median frequencies for each group. Paired statistical analysis was performed using Wilcoxon signed‐rank test and comparisons between groups were made using Mann–Whitney U test.*p < 0.05, **p < 0.01, ***p < 0.001,****p < 0.0001.

In vitro re‐stimulation of PBMC with TIV antigen revealed only limited induction of IFN‐γ and CD25 compared to the (background) response to adjuvanting low concentrations of IL‐12^+^ IL‐18 alone (Fig. 2A and C). Significant induction of NK cell CD107a expression in response to TIV was, however, observed at both baseline and all post‐vaccination time points, although there was no significant impact of vaccination (Fig. 2B). A similar pattern of responses was observed to antigen alone, in the absence of adjuvanting cytokines (data not shown). By comparison, NK cells from both HCMV seropositive and seronegative UK subjects made significant IFN‐γ and CD25 responses to TIV, although these were enhanced by vaccination only in HCMV seronegative donors (Fig. 2D and F). Moreover, both HCMV seropositive and seronegative UK donors made more potent CD107a responses to TIV than did the Gambian donors, although these were not enhanced after vaccination (Fig. 2E). None of the responses to TIV differed significantly between the different age groups of Gambian subjects, potentially reflecting the very early functional differentiation of NK cells in this population (Fig. 1) 28.

Similar patterns of NK‐cell responses occurred in Gambian adults vaccinated with DTPiP, with minimal NK‐cell responsiveness to DTPiP antigens either before or after vaccination (data not shown). Overall, these data are consistent with loss of antigen‐driven NK cell IFN‐γ responses in HCMV seropositive individuals while maintaining degranulation responses 6. There was limited induction of IL‐2 from CD4^+^ T cells, which could, in part explain the paucity of CD4^+^ T‐cell‐dependent NK‐cell responses on re‐stimulation with influenza antigen in vitro (Supporting Information Fig. 4).

### Impact of post‐vaccination antibody on vaccine antigen‐induced responses

Previous studies have shown a strong dependence of vaccine or influenza virus‐driven NK cell degranulation (and CD25 induction) on serum IgG antibodies 2, 6, 21, 22. We therefore tested sera collected after vaccination could promote or enhance vaccine‐antigen‐driven NK‐cell responses.

**Figure 3:**
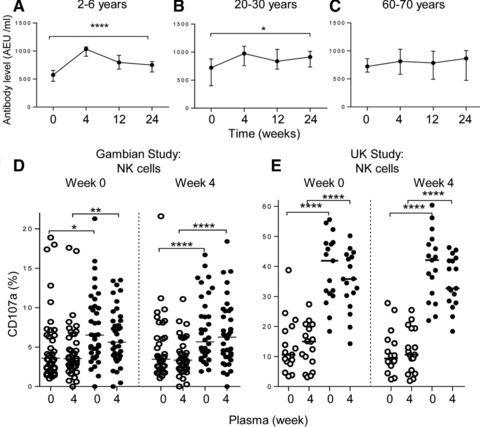
Vaccination does not enhance antibody‐dependent, TIV‐driven NK cell responses. (A‐C) Plasma IgG antibodies expressed as Arbitrary ELISA Units (AEU)/mL were measured by ELISA against TIV antigen for each age group. Dots indicate median antibody levels and bars represent IQR. Data are shown for 68 subjects. Repeated measures ANOVA was used to analyze the trend in antibody levels before and up to 24 weeks post‐vaccination *p < 0.05, ***p < 0.001; ****p < 0.0001. (D, E) Frequency of CD107a expressing NK cells at week 0 and week 4 post TIV vaccination of (D) Gambian and (E) UK donors. Cells were in medium alone (open symbols) or TIV antigen (closed symbols) in the presence of week 0 or week 4 autologous plasma. NK cells were gated as shown in Supporting Information Fig. 1H,K. Data are shown for 38 Gambian subjects, comprising 3 children, 17 adults and 18 elderly individuals (D) and for 17 TIV vaccinated UK adults (E). Paired statistical analysis was performed using Wilcoxon Signed rank test, *p < 0.05, **p < 0.01, ****p < 0.0001.

Anti‐TIV IgG concentrations were significantly boosted by vaccination in children (baseline, median 574 AEU/mL and interquartile range (IQR) 460–653; 4 weeks, 1033 and 908–1073) and in young adults (baseline, median 722 and 402–879; 4 weeks, 975 and 759–1104), whereas no significant increase was observed in older adults (baseline, median 723 and IQR 622–862; 4 weeks, 813 and 583–1031) (Fig. 3A–C). High median levels of IgG were present at baseline in all age groups, consistent with widespread natural exposure to influenza within the study population. Similar anti‐TIV specific antibody levels were observed in a TIV vaccinated UK cohort (baseline, median titre = 430, 299–767; 4 weeks, 962, 398–961). As shown for cells cultured in pooled control sera (Fig. 2), strong degranulation responses were observed in pre and post vaccination NK cells cultured with autologous plasma collected either at baseline or 4 weeks after vaccination (Fig. 3D). Similar observations were made in TIV vaccinated UK donors (Fig. 3E). However, no additional induction of CD107a expression was observed after vaccination, confirming that in both study cohorts, sufficient antibody was already present at baseline to drive these responses (Fig. 3D and E). TIV‐induced degranulation in Gambians was associated with the down‐regulation of CD16, which was further enhanced in post‐vaccination plasma, as previous described in UK donors 22 (median, IQR for MFI of CD16: Medium alone: Baseline plasma = 6539, 5137–7434; 4 weeks plasma = 5673, 5270–8823 and TIV: baseline = 1107, 810–1642; 4 weeks = 713, 562–1037, *p* = 0.0006).

### Vaccination enhances NK‐cell IFN‐γ responses to accessory cytokines

In UK subjects, influenza vaccination induces intrinsic changes in NK cells characteristic of “pre‐activated” NK cells; IFN‐γ and degranulation responses to exogenous cytokines were enhanced after vaccination 2.

**Figure 4:**
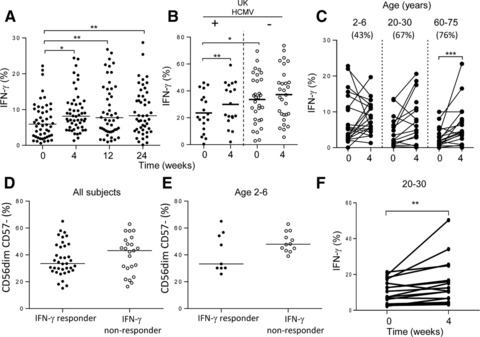
NK cell IFN‐γ responses to exogenous cytokines are significantly enhanced post vaccination. (A‐C) Frequencies of NK cells expressing IFN‐γ after restimulation with IL‐12 + IL‐18 (HCC) before and after vaccination with TIV in (A) Gambian and (B) UK donors. (C) Age‐stratified (2–6, 20–30, 60–75 years) NK cell IFN‐γ responses of Gambians to cytokines at baseline and 4 weeks after TIV vaccination showing the percentage of individuals within each age group with enhanced responses post‐vaccination. (D, E) Frequencies of baseline CD57‐ NK cells across the entire cohort (D) or in children only (E), split according to ‘responder’ individuals, with increased HCC driven IFN‐γ responses and ‘non‐responder’. (F) Responses to HCC in Gambian adults before and 4 weeks post‐vaccination with DTPiP. All NK cells analysis was gated as shown in Supporting Information Fig. 1. Data from 65 subjects are shown for TIV vaccinated Gambians (A, C), and 52 UK donors (comprising 19 HCMV+, closed symbols and 33 HCMV‐ individuals, open symbols) and from 18 Gambian subjects for DTPiP vaccination (F). Dots represent the frequency of (A,B,C,F) IFN‐γ^+^ or (C, D) CD57^−^ NK cells for each subject and horizontal lines represent median frequency for each group. Statistical analysis of paired samples (A, C, F) was performed using Wilcoxon signed‐rank test and for nonpaired analysis with Mann–Whitney U test, *p < 0.05, **p < 0.01.

To assess whether enhancement of these responses also occurred after TIV or DTPiP vaccination of Gambians with life‐long HCMV infection, pre and post‐vaccination PBMC were cultured with high concentrations of IL‐12 (5 ng/mL) plus IL‐18 (50 ng/mL) (HCC). Significant enhancement of NK‐cell IFN‐γ responses to HCC were observed by 4 weeks after TIV vaccination, which was stable and detectable for at least 24 weeks (Fig. 4A). By comparison, the responses of UK donors were only enhanced after vaccination for HCMV+ individuals, who had significantly lower baseline HCC‐stimulated IFN‐γ production compared to HCMV‐ individuals (Fig. 4B). In Gambians, this effect was highly significant among 60–75 year old individuals (Fig. 4C). 9 of 21 individuals within the 2–6 year old age group showed increased cytokine‐driven responses after vaccination and were categorized as vaccine ‘responder’ (median baseline IFN‐γ production: 2.8%, range 0–11%; 4 weeks: 8.3%, range 3.33‐19.7%). In contrast, ‘non‐responder’ children showing no increase in IFN‐γ production had high baseline production (median 10.9%, range 2.04‐22.8%) which tended to decrease after vaccination (median 6.0%, range 1.3‐13.3%). Comparing the distribution of NK cell subsets at baseline between these vaccine ‘responder’ and ‘non‐responder’ groups, individuals where boosting of the HCC driven IFN‐γ response was observed after vaccination tended to have lower frequencies of CD57‐ cells at baseline, a trend also observed for children alone (Fig. 4D and E).

Enhancement of responses to exogenous cytokines also occurred 4 weeks after DTPiP booster vaccination in adults (Fig. 4F). In contrast to previous observations in UK donors, IL‐12/IL‐18‐induced NK cell CD107a or CD25 expression was not enhanced after either TIV or DTPiP vaccination (Supporting Information Fig. 5A–D).

### Vaccination‐induced enhancement of IFN‐γ production among CD56^bright^ and CD56^dim^CD57^−^ subsets

**Figure 5:**
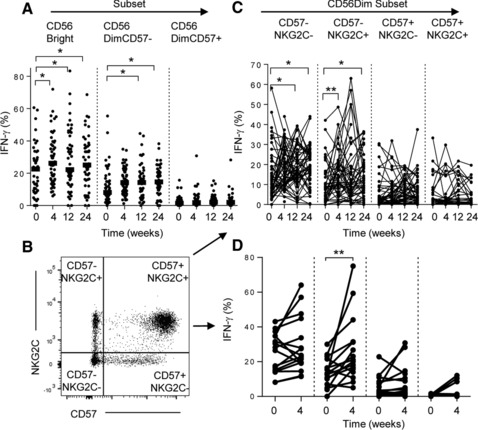
Enhancement of cytokine‐driven IFN‐γ production within CD56^bright^, CD56^dim^NKG2C^−^CD57^−^ and CD56^dim^NKG2C^+^CD57^−^ NK cells after vaccination. (A) Frequencies of IFN‐γ producing NK cells after HCC stimulation of PBMC at baseline (0) and up to 24 weeks post TIV vaccination. (B) Gating strategy showing CD57 and NKG2C‐defined subsets. (C, D) Subset distribution for NK cell IFN‐γ production after HCC stimulation of pre (0 weeks) and post‐vaccination (4, 12, 24 weeks) samples from subjects receiving (C) TIV and (D) DTPiP. Dots represent the frequency of IFN‐γ^+^ NK cells for each subject and horizontal lines represent median frequency for each group. Statistical analysis was performed on paired samples using Wilcoxon signed‐rank test, *p < 0.05, **p < 0.01.

As cytokine responsiveness varies by NK‐cell differentiation status 20, we analyzed the distribution of vaccine‐enhanced responses within NK‐cell subsets. As expected, IFN‐γ producing cells were more frequent in the CD56^bright^ NK cell subset than in the CD56^dim^CD57^+^ NK cell subset, with intermediate frequencies in CD56^dim^CD57^−^ cells (Fig. 5A). Moreover, the proportion of cells producing IFN‐γ in response to IL‐12/IL‐18 was significantly enhanced 4 weeks post‐vaccination in both the CD56^bright^ and the CD56^dim^CD57^−^ subsets but not in the CD56^dim^CD57^+^ subset (Fig. 5A). Strikingly, when grouping NK cells according to expression of CD57 and/or NKG2C, only the CD57^−^NKG2C^−^ and CD57^−^NKG2C^+^ CD56^dim^ demonstrated increased frequencies of IFN‐γ producing cells after vaccination (4 weeks, *p* = 0.004) which persisted up to 24 weeks (Fig. 5C). This enhancement was not due to enrichment of CD57/NKG2C subsets, since no change was observed in the proportions of CD56, CD57 and NKG2C‐defined subsets after vaccination (Supporting Information Fig. 5F). Significant enhancement of cytokine‐driven IFN‐γ responses was also observed within CD57‐ (NKG2C‐ or NKG2C+) NK cells for the 2–6 and 60–75 year old age groups (Supporting Information Fig. 6). Consistent with the effects of TIV, DTPiP booster vaccination of adults resulted in significant enhancement of the IFN‐γ response to rIL‐12/IL‐18 among CD56^dim^CD57^−^NKG2C^+^ NK cells (*p* = 0.009, 4 weeks) and there was a trend for enhanced responsiveness among CD56^dim^CD57^−^NKG2C^−^ cells (Fig. 5D).

## Discussion

In contrast to the enhancement of CD4 T‐cell‐dependent NK‐cell responses to vaccine antigens in previous studies of UK vaccinees 2, 5, we consistently failed to detect TIV or DTPiP vaccine antigen‐driven NK cell IFN‐γ or CD25 responses in Gambian donors. Although antigen‐driven CD107a responses were detected, these were not boosted by vaccination. The very high prevalence of HCMV among the Gambian population may partially explain these observations since augmentation of NK cell responses by vaccination was significantly more pronounced among HCMV‐ than among HCMV^+^ UK subjects 2. Nonetheless, the IFN‐γ and CD25 responses to vaccine antigens among NK cells from Gambian donors are typically much lower than those of HCMV^+^ UK donors, suggesting that HCMV infection status alone does not fully explain the lack of response after vaccination. In addition, we have previously observed NK cell phenotypical and functional discrepancies between HCMV^+^ Gambian and UK donors (23). Further studies are required to determine whether differences in the timing of HCMV infection, differences in strains of infecting virus, or other factors including co‐infections, might explain this lack of vaccine antigen‐driven NK function in Gambians of all ages.

Despite the lack of “boosting” of antigen‐induced NK cell responses after vaccination in Gambian subjects, this study provides further evidence for enhancement of cytokine‐driven NK cell IFN‐γ responses. TIV, DTPiP and likely other vaccines may enhance these responses through a common mechanism, with similar mechanisms potentially underlying protracted enhancement of NK cell responses after BCG booster vaccination in other African populations 39 and in European populations after yellow fever vaccination 40. In this study, such vaccine‐stimulated NK cells persist for at least 6 months whereas in our UK‐based studies, these effects were lost by 3 months. Possible explanations for this difference could be that responses in Gambian individuals are boosted by more frequent exposures to environmental pathogens or that the relative stronger responses of both HCMV^−^ and HCMV^+^ UK donors are less amenable to sustained enhancement.

Restriction of this effect to the CD56^bright^, CD56^dim^CD57^−^NKG2C^−^ and CD56^dim^CD57^−^NKG2C^+^ subset of NK cells is reminiscent of the less differentiated NK cells generated in vitro by cytokine pre‐activation 14, 41. Heightened cytokine‐driven IFN‐γ responses within CD56^dim^CD57^−^NKG2C^−/+^ NK cells also suggests that these cells are equivalent to the less differentiated CD57^−^ cells observed in UK donors with enhanced cytokine sensitivity and higher intrinsic proliferative capacity (Ki67) after influenza vaccination 2. These data are also consistent with the acquisition of NKG2C, and expansion of NKG2C^+^ cells, prior to functional differentiation and CD57 acquisition 28. Interestingly, TIV vaccinated individuals showing enhanced responses to cytokines after vaccination tended to have fewer cytokine‐driven IFN‐γ^+^ NK cells and of CD56^dim^CD57^−^ cells at baseline compared to those with no boosting. Differences in cytokine driven responses observed at baseline were, however, not attributable to ongoing environmental background activation as we saw no differences in ex‐vivo expression of CD25, CD107a or IFN‐γ comparing ‘responder’ and ‘non‐responder’ groups. This pattern of responses parallels that observed in UK subjects where enhancement of cytokine driven responses after TIV vaccination occurs principally in HCMV^+^ individuals with suboptimal baseline responses and not in HCMV‐ individuals, who already have maximal frequencies of IFN‐γ^+^ cells 2.

Differentiation subsets of NK cells could have varying thresholds of cytokine (Interleukins‐2, 12, 15, and 18) responsiveness, thereby influencing the effective potency of the different cytokines induced by vaccination. The acquisition of CD57 correlates with decreased expression of IL‐12Rβ2 and IL‐18Rα 20. However, when comparing HCMV^+^ to HCMV^−^ individuals we have observed lower responsiveness within each CD57 defined subset, which cannot be explained by cytokine receptor expression alone 2, 24.

Vaccine‐induced enhancement of IFN‐γ secretion in less differentiated NK cells could reflect preferential proliferative expansion, epigenetic modifications or a combination of both of these processes. Epigenetic modifications have been demonstrated in human and murine NK cells after cytokine pre‐activation which, in turn, promote increased IFN‐γ transcription 42, 43. One of the limitations of this study is that, we had insufficient available paired pre and post‐vaccination cells investigate whether CD56^dim^CD57/NKG2C^+^ cells are particularly sensitive to vaccine induced epigenetic modifications. Examination of other NK cell cytokines such as tumor necrosis factor‐α and granulocyte‐macrophage colony‐stimulating factor would also reveal whether vaccine‐induced enhancement is specific for IFN‐γ production.

In summary, two very different vaccines are able to re‐set the threshold for human NK cell IFN‐γ responses to cytokines (in an antigen‐independent manner) in individuals where HCMV has had a major effect on NK‐cell functional differentiation. Enhancement of cytokine‐driven NK‐cell IFN‐γ responses is particularly evident in elderly individuals and within a distinct population of CD56^dim^CD57^−^NKG2C^+^ NK cells that accumulate in HCMV‐infected individuals. This phenomenon may partially compensate for the terminal differentiation of NK cells, away from cytokine responsiveness, observed in HCMV seropositive donors 3, 11, 25, 26, 28 and may also contribute to the expansion of NKG2C^+^ NK cell populations that is observed among HCMV‐infected subjects after systemic viral infections 44, 45, 46, 47. Vaccine‐driven enhancement of effector functions in CD56^dim^CD57^−^NKG2C^−^ and CD56^dim^CD57^−^NKG2C^+^ NK merits further investigation as a route to improve vaccine efficacy in HCMV infected elderly individuals.

## Materials and methods

### Study subjects

The Medical Research Council Gambia (MRCG) Scientific Coordinating Committee (SCC), The Gambia Government and MRCG Joint Ethics Committee (SCC reference 1309), London School of Hygiene and Tropical Medicines Research Ethics Committee (LSHTM reference 6237 and 6331) and The Republic of The Gambia Medicines Board approved this study.

In order to minimize the impact of natural exposure to influenza during the follow‐up period, sample collection times fell outside the main Gambian influenza season 48. Following written informed consent, 68 healthy participants were recruited from three villages (Keneba, Manduar, and Kantong Kunda) in West Kiang, The Gambia. Participants fell into one of three age groups: 2–6, 20–30 and 60–75 years of age. Individuals with chronic disease, infections, or influenza‐like signs and symptoms during the previous 3 months or with an axillary temperature of ≥ 38°C were excluded. Pregnant women, individuals potentially allergic to vaccine products, and those with a history of previous influenza vaccination were also excluded. Venous blood was collected at baseline (pre‐vaccination) and participants were vaccinated intramuscularly with 2012–2013 northern hemisphere, seasonal, trivalent inactivated influenza split virion vaccine (Influenza A/California/7/2009 (H1N1) pdm09‐like virus; Influenza A/Victoria/361/2011 (H3N2)‐like virus; and Influenza B/Wisconsin/1/2010‐like virus; Sanofi Pasteur MSD). Subsequent blood samples were collected 1, 3, and 6 month(s) post‐vaccination.

For vaccination with tetravalent DTPiP, 18 adult males between the ages of 21 and 29 years (median age = 24.2 years) were recruited. All subjects previously received DTP and oral polio vaccine as children and had not since been boosted. This study was conducted in Sukuta, West Coast Region, The Gambia. A single dose of DTPiP (Repevax™ Sanofi Pasteur MSD) was given intramuscularly after collecting a baseline blood sample. A follow‐up blood sample was collected 4 weeks later.

### Serology

Baseline samples from the influenza study were screened for IgG antibodies to HCMV (Biokit, Barcelona, Spain) and IgG antibody against Epstein Barr virus (EBV) nuclear antigen‐1 (EBNA‐1) (Euroimmun, Lubeck, Germany). Seropositivity was determined according to HCMV‐ and EBV‐negative standards provided by the manufacturer. Influenza vaccine antigen‐specific IgG was determined by an in‐house ELISA as previously described 2, 6.

### Cell preparation and culture

Peripheral Blood mononuclear cells (PBMC) were prepared by centrifugation of whole blood over Lymphoprep^TM^ (Sigma, UK). PBMC were reserved for ex vivo staining or cryo‐preserved in freezing medium (5% DMSO in Fetal Calf Serum, FCS) at ‐80°C (1 × 10^7^ PBMC per vial) overnight in Mr. Frosty™ Freezing Containers, prior to transfer into liquid nitrogen. Cells were recovered by rapid thawing into culture medium and repeated washing, and rested for 3 h prior to stimulation. Viability of recovered cells was >95% and yields ranged from 30 to 65%.

In vitro restimulation assays were performed simultaneously on cells from all pre and post vaccination time points for a given individual. PBMC (3 × 10^5^/ well) were cultured for 18 h in 96‐well U‐bottom plates (Costar^®^ cell culture plates, USA) at 37°C, 5% CO_2._ For T‐cell assays, cells were stimulated for a total of 5 h. TIV and DTPiP vaccine antigens were used at final concentrations of 2.5 and 0.5 μg/mL, respectively. Low Concentration of Cytokines (LCC) comprised 12.5 pg/mL recombinant rIL‐12 (Peprotech, London, UK) + 10 ng/mL rIL‐18 (R&D systems, Abingdon, UK). High Concentration of Cytokine (HCC) comprised 5 ng/mL rIL‐12 + 50 ng/mL rIL‐18. GolgiStop, containing Monensin and GolgiPlug, containing Brefeldin A (both from BD Biosciences, Oxford, UK) were added after 2 h for T cell and after 15 h for NK‐cell assays. Culture medium was RPMI 1640 supplemented with Penicillin‐Streptomycin‐Glutamine (Gibco, UK) and 10% pooled human male AB serum (Sigma‐Aldrich®, Saint Louis, USA) unless otherwise stated.

### Flow cytometric analysis

For ex vivo antibody staining, cells plated at 3 × 10^5^ cells/well in a 96‐well U‐bottom plate. NK cell phenotype and function was determined using CD56‐PE‐Cy7 (Clone NCAM‐16.2), CD3‐V500 (Clone UCHT1), CD107a‐FITC (H4A3), (all from BD Biosciences, Oxford UK); NKG2C‐PE (134591, R&D Systems, UK), CD25‐PerCP‐Cy5.5 (BC96), CD57‐e450 (TB01, both from eBioscience/Affimetrix, UK), NKG2A‐APC (Clone Z199, Beckman Coulter, UK). Anti‐CD107a antibody was added at the initiation of cell cultures as previously described 49. T cell differentiation and function were determined using combinations of CD3‐V500, CD45RA‐APC‐H7 (Clone HI100, BD Biosciences), CD4‐PE (Clone OKT4), CD8‐PerCP‐Cy5.5 (Clone RPA‐T8), CD27‐FITC (Clone O323), CD28‐PE‐Cy7 (Clone CD28.2), CD57‐e450 (All from eBiosciences) and CCR7‐APC (150503, R&D systems). The relative frequencies of T cells, B cells, monocytes, and myeloid/ plasmocytoid dendritic cells were determined using CD56‐PE‐Cy7, CD3‐V500 (Both from BD Biosciences), CD45‐FITC (Clone HI30), CD11c‐PE (Clone 3.9), CD19‐PerCP‐Cy5.5 (Clone HIB19), CD123‐e450 (Clone 6H6), CD14‐APC‐e780 (Clone 61D3) (all from eBioscience/ Affimetrix). Intracellular staining of NK cells and T cells was performed after fixation and permeablisation (BD Bioscience) and addition of IFN‐γ‐APC‐e780 (Clone 4SB3, eBiosciences) or anti‐IFN‐γ APC (Clone B27, BD Biosciences) and anti‐IL‐2 PE (Clone MQ17‐H12, BD Biosciences), respectively. Samples were acquired on a Cyan ADP flow cytometer with Summit® software (influenza vaccine study), or LSRII or LSRIII Fortessa flow cytometer with FacsDiva® software (DTPiP vaccine study). All FACS data analyses were performed using FlowJo® (TreeStar). Where less than 100 events were obtained within a gated NK cell subset, that sample was excluded from the analysis.

### Statistical analysis

Nonparametric Wilcoxon matched paired tests were used for intra‐group comparisons between pre and post vaccination data. Kruskal–Wallis tests were used for unpaired comparisons between age groups, for nonpaired analysis Mann–Whitney U test was used. Linear trend analyses were performed using repeated measures ANOVA. GraphPad Prism (GraphPad Software 6) was used for statistical analysis. Significant differences are reported as *p < 0.05, **p < 0.01, ***p < 0.001, ****p < 0.0001.

## Conflict of interest

The authors declare no financial or commercial conflict of interest.

**Abbreviations**
NK: natural killer
IL‐2: interleukin‐2
TIV: trivalent influenza vaccine
DTPiP: diptheria, tetanus, pertussis and inactivated polio vaccine
ADCC: antibody dependent cellular cytotoxicity
HCC: high concentration of cytokines
AEU: arbitrary ELISA units
NCR: natural cytotoxicity receptor
HCMV: human cytomegalovirus

## Supporting information

Supporting Figures

Peer review correspondence

## References

[1] Kiessling, R. , Klein, E. , Pross, H. and Wigzell, H. , “Natural” killer cells in the mouse. II. Cytotoxic cells with specificity for mouse Moloney leukemia cells. Characteristics of the killer cell. Eur. J. Immunol. 1975 5: 117–121. 1086218 10.1002/eji.1830050209

[2] Goodier, M. R. , Rodriguez‐Galan, A. , Lusa, C. , Nielsen, C. M. , Darboe, A. , Moldoveanu, A. L. , White, M. J. **et al**, Influenza vaccination generates cytokine‐induced memory‐like NK cells: impact of human cytomegalovirus infection. J. Immunol. 2016 197: 313–325. 27233958 10.4049/jimmunol.1502049 PMC4911617

[3] Lee, J. , Zhang, T. , Hwang, I. , Kim, A. , Nitschke, L. , Kim, M. , Scott, J. M. **et al**, Epigenetic modification and antibody‐dependent expansion of memory‐like NK cells in human cytomegalovirus‐infected individuals. Immunity 2015 42: 431–442. 25786175 10.1016/j.immuni.2015.02.013 PMC4537797

[4] Fehniger, T. A. , Cooper, M. A. , Nuovo, G. J. , Cella, M. , Facchetti, F. , Colonna, M. and Caligiuri, M. A. , CD56bright natural killer cells are present in human lymph nodes and are activated by T cell‐derived IL‐2: a potential new link between adaptive and innate immunity. Blood 2003 101: 3052–3057. 12480696 10.1182/blood-2002-09-2876

[5] Horowitz, A. , Behrens, R. H. , Okell, L. , Fooks, A. R. and Riley, E. M. , NK cells as effectors of acquired immune responses: effector CD4+ T cell‐dependent activation of NK cells following vaccination. J. Immunol. 2010 185: 2808–2818. 20679529 10.4049/jimmunol.1000844

[6] Nielsen, C. M. , White, M. J. , Bottomley, C. , Lusa, C. , Rodriguez‐Galan, A. , Turner, S. E. , Goodier, M. R. **et al**, Impaired NK cell responses to pertussis and H1N1 influenza vaccine antigens in human cytomegalovirus‐infected individuals. J. Immunol. 2015 194: 4657–4667. 25855356 10.4049/jimmunol.1403080 PMC4416741

[7] Jost, S. , Tomezsko, P. J. , Rands, K. , Toth, I. , Lichterfeld, M. , Gandhi, R. T. and Altfeld, M. , CD4+ T‐cell help enhances NK cell function following therapeutic HIV‐1 vaccination. J. Virol. 2014 88: 8349–8354. 24829350 10.1128/JVI.00924-14 PMC4135926

[8] Vieira Braga, F. A. , Hertoghs, K. M. , van Lier, R. A. and van Gisbergen, K. P. , Molecular characterization of HCMV‐specific immune responses: parallels between CD8(+) T cells, CD4(+) T cells, and NK cells. Eur. J. Immunol. 2015 45: 2433–2445. 26228786 10.1002/eji.201545495

[9] Tesi, B. , Schlums, H. , Cichocki, F. and Bryceson, Y. T. , Epigenetic regulation of adaptive NK cell diversification. Trends Immunol. 2016 37: 451–461. 27160662 10.1016/j.it.2016.04.006

[10] Guma, M. , Budt, M. , Saez, A. , Brckalo, T. , Hengel, H. , Angulo, A. and Lopez‐Botet, M. , Expansion of CD94/NKG2C+ NK cells in response to human cytomegalovirus‐infected fibroblasts. Blood 2006 107: 3624–3631. Epub 2005 Dec 3629. 10.1182/blood-2005-09-3682 16384928

[11] Schlums, H. , Cichocki, F. , Tesi, B. , Theorell, J. , Beziat, V. , Holmes, T. D. , Han, H. **et al**, Cytomegalovirus infection drives adaptive epigenetic diversification of NK cells with altered signaling and effector function. Immunity 2015 42: 443–456. 25786176 10.1016/j.immuni.2015.02.008 PMC4612277

[12] O'Leary, J. G. , Goodarzi, M. , Drayton, D. L. and von Andrian, U. H. , T cell‐ and B cell‐independent adaptive immunity mediated by natural killer cells. Nat. Immunol. 2006 7: 507–516. Epub 2006 Apr 2016. 16617337 10.1038/ni1332

[13] Sun, J. C. , Beilke, J. N. and Lanier, L. L. , Adaptive immune features of natural killer cells. Nature 2009 457: 557–561. 19136945 10.1038/nature07665 PMC2674434

[14] Cooper, M. A. , Elliott, J. M. , Keyel, P. A. , Yang, L. , Carrero, J. A. and Yokoyama, W. M. , Cytokine‐induced memory‐like natural killer cells. Proc. Natl. Acad. Sci. USA 2009 106: 1915–1919. 19181844 10.1073/pnas.0813192106 PMC2644138

[15] Leong, J. W. , Chase, J. M. , Romee, R. , Schneider, S. E. , Sullivan, R. P. , Cooper, M. A. and Fehniger, T. A. , Preactivation with IL‐12, IL‐15, and IL‐18 induces CD25 and a functional high‐affinity IL‐2 receptor on human cytokine‐induced memory‐like natural killer cells. Biol. Blood. Marrow. Transplant. 2014 20: 463–473. 24434782 10.1016/j.bbmt.2014.01.006 PMC3959288

[16] Guma, M. , Angulo, A. , Vilches, C. , Gomez‐Lozano, N. , Malats, N. and Lopez‐Botet, M. , Imprint of human cytomegalovirus infection on the NK cell receptor repertoire. Blood 2004 104: 3664–3671. 15304389 10.1182/blood-2004-05-2058

[17] Hwang, I. , Zhang, T. , Scott, J. M. , Kim, A. R. , Lee, T. , Kakarla, T. , Kim, A. **et al**, Identification of human NK cells that are deficient for signaling adaptor FcRgamma and specialized for antibody‐dependent immune functions. Int. Immunol. 2012 24: 793–802. 22962434 10.1093/intimm/dxs080 PMC3621379

[18] Rolle, A. and Brodin, P. , Immune adaptation to environmental influence: the case of NK cells and HCMV. Trends Immunol. 2016 37: 233–243. 26869205 10.1016/j.it.2016.01.005

[19] Horowitz, A. , Hafalla, J. C. , King, E. , Lusingu, J. , Dekker, D. , Leach, A. , Moris, P. **et al**, Antigen‐specific IL‐2 secretion correlates with NK cell responses after immunization of Tanzanian children with the RTS,S/AS01 malaria vaccine. J. Immunol. 2012 188: 5054–5062. 22504653 10.4049/jimmunol.1102710 PMC3378032

[20] White, M. J. , Nielsen, C. M. , McGregor, R. H. M. , Riley, E. M. and Goodier, M. R. , Differential activation of CD57‐defined natural killer cell subsets during recall responses to vaccine antigens. Immunology 2014 142: 140–150. 24843874 10.1111/imm.12239 PMC3992055

[21] Jegaskanda, S. , Luke, C. , Hickman, H. D. , Sangster, M. Y. , Wieland‐Alter, W. F. , McBride, J. M. , Yewdell, J. W. **et al**, Generation and protective ability of influenza virus‐specific antibody‐dependent cellular cytotoxicity in humans elicited by vaccination, natural infection, and experimental challenge. J. Infect. Dis. 2016 214: 945–952. 27354365 10.1093/infdis/jiw262 PMC4996149

[22] Goodier, M. , Lusa, C. , Sherratt, S. , Rodriguez‐Galan, A. , Behrens, R. and Riley, E. , Sustained immune complex‐mediated reduction in CD16 expression after vaccination regulates NK cell function. Front. Immunol. 2016 7: 1–13, article 384. 27725819 10.3389/fimmu.2016.00384 PMC5035824

[23] Jegaskanda, S. , Job, E. R. , Kramski, M. , Laurie, K. , Isitman, G. , de Rose, R. , Winnall, W. R. **et al**, Cross‐reactive influenza‐specific antibody‐dependent cellular cytotoxicity antibodies in the absence of neutralizing antibodies. J. Immunol. 2013 190: 1837–1848. 23319732 10.4049/jimmunol.1201574

[24] Nielsen, C. M. , Wolf, A. S. , Goodier, M. R. and Riley, E. M. , Synergy between Common gamma chain family cytokines and IL‐18 potentiates innate and adaptive pathways of NK cell activation. Front. Immunol. 2016 7: 101. 27047490 10.3389/fimmu.2016.00101 PMC4801862

[25] Lopez‐Verges, S. , Milush, J. M. , Pandey, S. , York, V. A. , Arakawa‐Hoyt, J. , Pircher, H. , Norris, P. J. **et al**, CD57 defines a functionally distinct population of mature NK cells in the human CD56dimCD16+ NK‐cell subset. Blood 2010 116: 3865–3874. 20733159 10.1182/blood-2010-04-282301 PMC2981540

[26] Bjorkstrom, N. K. , Riese, P. , Heuts, F. , Andersson, S. , Fauriat, C. , Ivarsson, M. A. , Bjorklund, A. T. **et al**, Expression patterns of NKG2A, KIR, and CD57 define a process of CD56dim NK‐cell differentiation uncoupled from NK‐cell education. Blood 2010 116: 3853–3864. 20696944 10.1182/blood-2010-04-281675

[27] Nielsen, C. M. , White, M. J. , Goodier, M. R. and Riley, E. M. , Functional significance of CD57 expression on human NK cells and relevance to disease. Front. Immunol. 2013 4: 422. 24367364 10.3389/fimmu.2013.00422 PMC3856678

[28] Goodier, M. R. , White, M. J. , Darboe, A. , Nielsen, C. M. , Goncalves, A. , Bottomley, C. , Moore, S. E. **et al**, Rapid NK cell differentiation in a population with near‐universal human cytomegalovirus infection is attenuated by NKG2C deletions. Blood 2014 124: 2213–2222. 25150297 10.1182/blood-2014-05-576124 PMC4206953

[29] Beziat, V. , Liu, L. L. , Malmberg, J. A. , Ivarsson, M. A. , Sohlberg, E. , Bjorklund, A. T. , Retiere, C. **et al**, NK cell responses to cytomegalovirus infection lead to stable imprints in the human KIR repertoire and involve activating KIRs. Blood 2013 121: 2678–2688. 23325834 10.1182/blood-2012-10-459545 PMC3617633

[30] Prod'homme, V. , Tomasec, P. , Cunningham, C. , Lemberg, M. K. , Stanton, R. J. , McSharry, B. P. , Wang, E. C. **et al**, Human cytomegalovirus UL40 signal peptide regulates cell surface expression of the NK cell ligands HLA‐E and gpUL18. J. Immunol. 2012 188: 2794–2804. 22345649 10.4049/jimmunol.1102068 PMC3303119

[31] Guma, M. , Cabrera, C. , Erkizia, I. , Bofill, M. , Clotet, B. , Ruiz, L. and Lopez‐Botet, M. , Human cytomegalovirus infection is associated with increased proportions of NK cells that express the CD94/NKG2C receptor in aviremic HIV‐1‐positive patients. J. Infect. Dis. 2006 194: 38–41. 16741880 10.1086/504719

[32] Adland, E. , Klenerman, P. , Goulder, P. and Matthews, P. C. , Ongoing burden of disease and mortality from HIV/CMV coinfection in Africa in the antiretroviral therapy era. Front. Microbiol. 2015 6: 1016. 26441939 10.3389/fmicb.2015.01016 PMC4585099

[33] Manicklal, S. , Emery, V. C. , Lazzarotto, T. , Boppana, S. B. and Gupta, R. K. , The "silent" global burden of congenital cytomegalovirus. Clin. Microbiol. Rev. 2013 26: 86–102. 23297260 10.1128/CMR.00062-12 PMC3553672

[34] Romee, R. , Schneider, S. E. , Leong, J. W. , Chase, J. M. , Keppel, C. R. , Sullivan, R. P. , Cooper, M. A. **et al**, Cytokine activation induces human memory‐like NK cells. Blood 2012 120: 4751–4760. 22983442 10.1182/blood-2012-04-419283 PMC3520618

[35] Goncalves, A. , Makalo, P. , Joof, H. , Burr, S. , Ramadhani, A. , Massae, P. , Malisa, A. **et al**, Differential frequency of NKG2C/KLRC2 deletion in distinct African populations and susceptibility to Trachoma: a new method for imputation of KLRC2 genotypes from SNP genotyping data. Hum. Genet. 2016 135: 939–951. 27312142 10.1007/s00439-016-1694-2 PMC4947484

[36] Palmer, B. E. , Blyveis, N. , Fontenot, A. P. and Wilson, C. C. , Functional and phenotypic characterization of CD57+CD4+ T cells and their association with HIV‐1‐induced T cell dysfunction. J. Immunol. 2005 175: 8415–8423. 16339584 10.4049/jimmunol.175.12.8415

[37] Koch, S. , Larbi, A. , Derhovanessian, E. , Ozcelik, D. , Naumova, E. and Pawelec, G. , Multiparameter flow cytometric analysis of CD4 and CD8 T cell subsets in young and old people. Immun. Ageing. 2008 5: 6. 18657274 10.1186/1742-4933-5-6 PMC2515281

[38] Strioga, M. , Pasukoniene, V. and Characiejus, D. , CD8+ CD28‐ and CD8+ CD57+ T cells and their role in health and disease. Immunology 2011 134: 17–32. 21711350 10.1111/j.1365-2567.2011.03470.x PMC3173691

[39] Suliman, S. , Geldenhuys, H. , Johnson, J. L. , Hughes, J. E. , Smit, E. , Murphy, M. , Toefy, A. **et al**, Bacillus Calmette‐Guerin (BCG) revaccination of adults with latent Mycobacterium tuberculosis infection induces long‐lived BCG‐reactive NK cell responses. J. Immunol. 2016 197: 1100–1110. 27412415 10.4049/jimmunol.1501996 PMC4976036

[40] Marquardt, N. , Ivarsson, M. A. , Blom, K. , Gonzalez, V. D. , Braun, M. , Falconer, K. , Gustafsson, R. **et al**, The human NK cell response to yellow fever virus 17D is primarily governed by NK cell differentiation independently of NK cell education. J. Immunol. 2015 195: 3262–3272. 26283480 10.4049/jimmunol.1401811

[41] Berrien‐Elliott, M. M. , Wagner, J. A. and Fehniger, T. A. , Human cytokine‐induced memory‐like natural killer cells. J. Innate Immun. 2015 7: 563–571. 25924651 10.1159/000382019 PMC4843115

[42] Luetke‐Eversloh, M. , Hammer, Q. , Durek, P. , Nordstrom, K. , Gasparoni, G. , Pink, M. , Hamann, A. **et al**, Human cytomegalovirus drives epigenetic imprinting of the IFNG locus in NKG2Chi natural killer cells. PLoS Pathog. 2014 10: e1004441. 25329659 10.1371/journal.ppat.1004441 PMC4199780

[43] Ni, J. , Holsken, O. , Miller, M. , Hammer, Q. , Luetke‐Eversloh, M. , Romagnani, C. and Cerwenka, A. , Adoptively transferred natural killer cells maintain long‐term antitumor activity by epigenetic imprinting and CD4+ T cell help. Oncoimmunology 2016 5: e1219009. 27757318 10.1080/2162402X.2016.1219009 PMC5048776

[44] Beziat, V. , Dalgard, O. , Asselah, T. , Halfon, P. , Bedossa, P. , Boudifa, A. , Hervier, B. **et al**, CMV drives clonal expansion of NKG2C+ NK cells expressing self‐specific KIRs in chronic hepatitis patients. Eur. J. Immunol. 2012 42: 447–457. 22105371 10.1002/eji.201141826

[45] Bjorkstrom, N. K. , Lindgren, T. , Stoltz, M. , Fauriat, C. , Braun, M. , Evander, M. , Michaelsson, J. **et al**, Rapid expansion and long‐term persistence of elevated NK cell numbers in humans infected with hantavirus. J. Exp. Med. 2011 208: 13–21. 21173105 10.1084/jem.20100762 PMC3023129

[46] Petitdemange, C. , Becquart, P. , Wauquier, N. , Beziat, V. , Debre, P. , Leroy, E. M. and Vieillard, V. , Unconventional repertoire profile is imprinted during acute chikungunya infection for natural killer cells polarization toward cytotoxicity. PLoS Pathog. 2011 7: e1002268. 21966274 10.1371/journal.ppat.1002268 PMC3178577

[47] Saghafian‐Hedengren, S. , Sohlberg, E. , Theorell, J. , Carvalho‐Queiroz, C. , Nagy, N. , Persson, J. O. , Nilsson, C. **et al**, Epstein‐Barr virus coinfection in children boosts cytomegalovirus‐induced differentiation of natural killer cells. J. Virol. 2013 87: 13446–13455. 24089567 10.1128/JVI.02382-13 PMC3838261

[48] World Health Organization , Influenza transmission zone:Western Africa 2012.

[49] Alter, G. , Malenfant, J. M. and Altfeld, M. , CD107a as a functional marker for the identification of natural killer cell activity. J. Immunol. Methods 2004 294: 15–22. 15604012 10.1016/j.jim.2004.08.008

