# Supplementary material for: Enhancement of cytokine‐driven NK cell IFN‐γ production after vaccination of HCMV infected Africans
Source: Eur J Immunol. 2017 Apr 24;47(6):1040–50. doi: 10.1002/eji.201746974 (PMC5888140; doi:10.1002/eji.201746974)
Supplement: Supplementary file 1 — Supporting Figures [file EJI-47-1040-s001.pdf]

European Journal of Immunology

Supporting Information
for
DOI 10.1002/eji.201746974

Alansana Darboe, Ebrima Danso, Ed Clarke, Ama Umesi, Ebrima Touray, Rita Wegmuller, Sophie E. Moore, Eleanor M. Riley and Martin R. Goodier

Enhancement of cytokine-driven NK cell IFN-γ production after vaccination of HCMV infected Africans

© 2017 The Authors. *European Journal of Immunology* published by WILEY-VCH Verlag GmbH & Co. KGaA, Weinheim.

Supporting Fig.1.

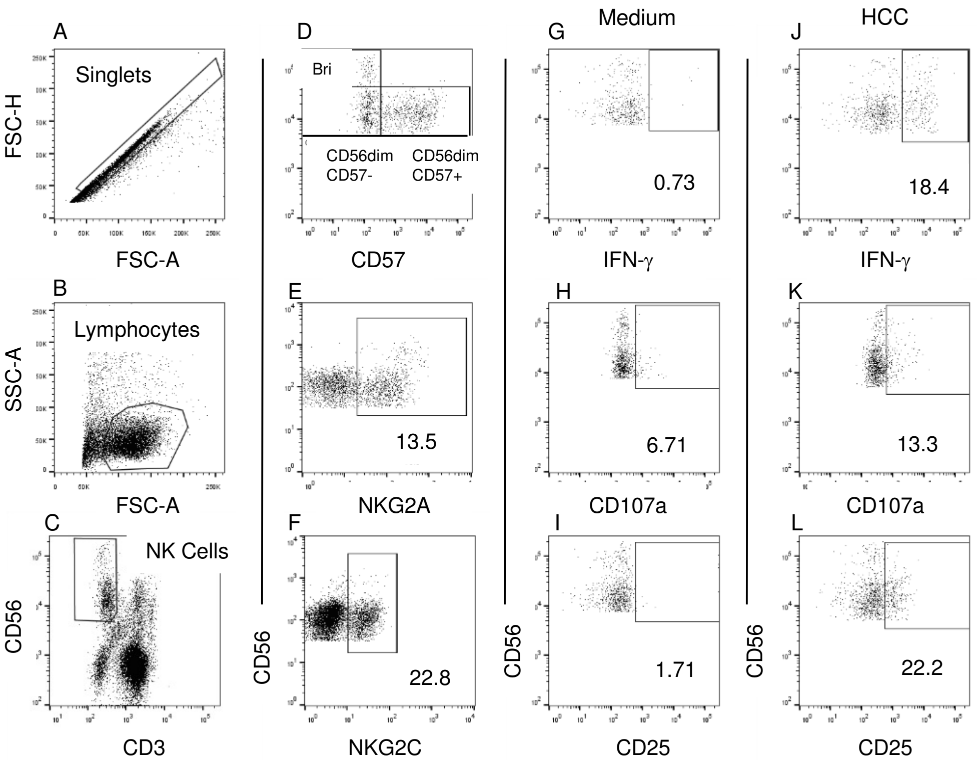

Supporting Fig.1. Gating strategy for functional NK cell subsets. (A) Singlet gate, (B) Lymphocyte gate, (C) $CD3^-CD56^+$ NK cell gate, (D) CD57-defined NK subsets ($CD56^{bright}$; $CD56^{dim}CD57^-$; $CD56^{dim}CD57^+$). Receptor expression and functional markers are shown within $CD3^-$ $CD56^+$ NK cells: (E) ex vivo $NKG2A^+$ expression and (F) $NKG2C^+$ expression; in vitro (G) IFN-g^+ (H) $CD107a^+$ and (I) $CD25^+$ responses in medium alone; (J) IFN-γ^+ (K) $CD107a^+$ and (L) $CD25^+$ responses to high concentration of cytokines (HCC).

Supporting Fig.2.

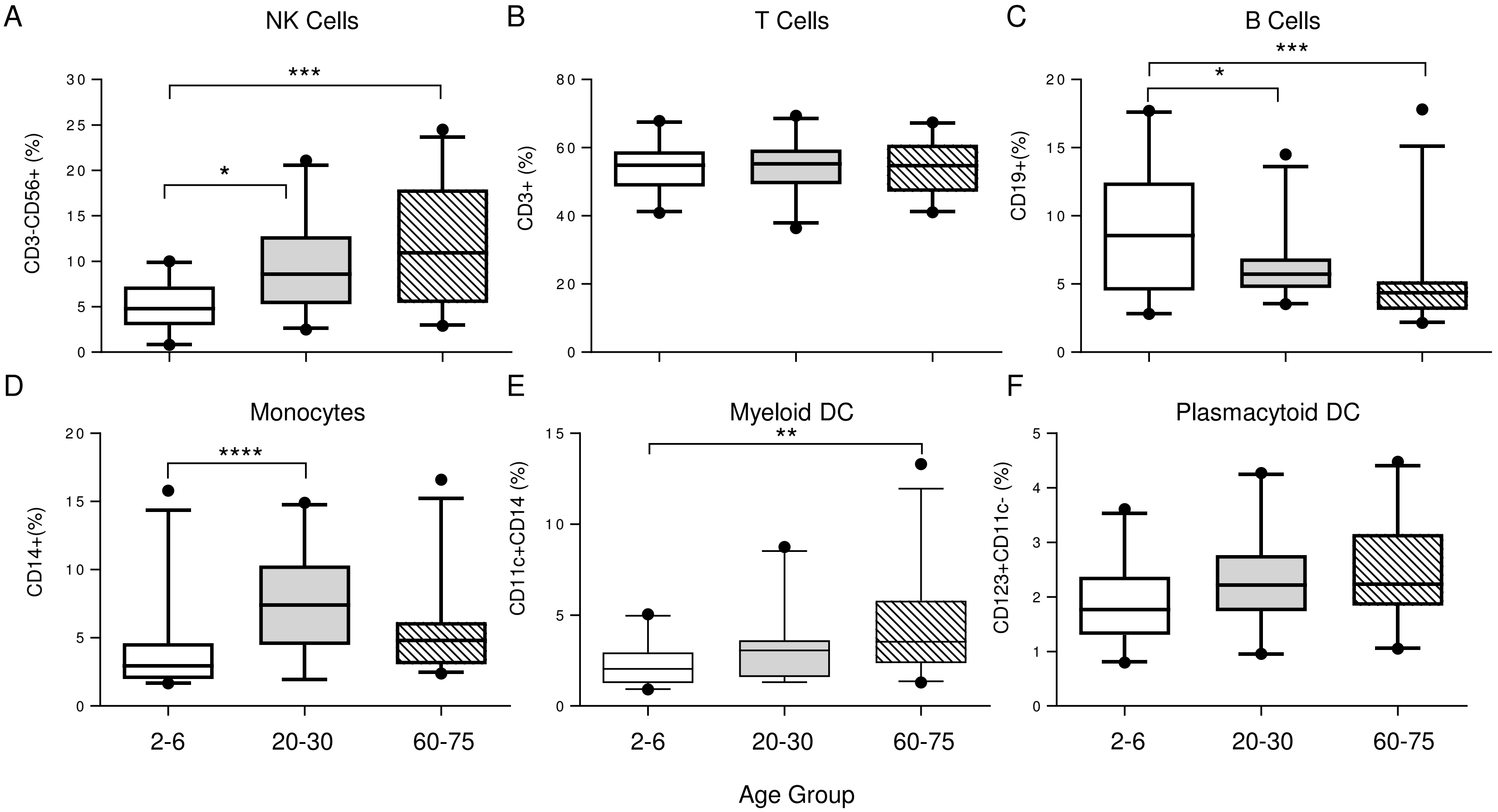

Supporting Fig.2. Variation in leukocyte populations with age. Proportions of different leukocyte populations at baseline among the three age groups (2-6, 20-30, 60-75 years), gated from $CD45^+$ cells. These cells were stained ex vivo without stimulation. (A) NK cells (B) T cells (C) B cells (D) monocytes (E) myeloid dendritic cells (F) plasmacytoid dendritic cells. Data are shown for 68 subjects. In box and whisker plots, the horizontal bar indicates median frequency, the boxes extend 25th-75th percentile range and the whiskers indicate 95th percentiles. Statistical analysis was performed on samples using Kruskal-Wallis test, *$p<0.05$, **$p<0.01$, ***$p<0.001$, ****$p<0.0001$.

Supporting Fig.3.

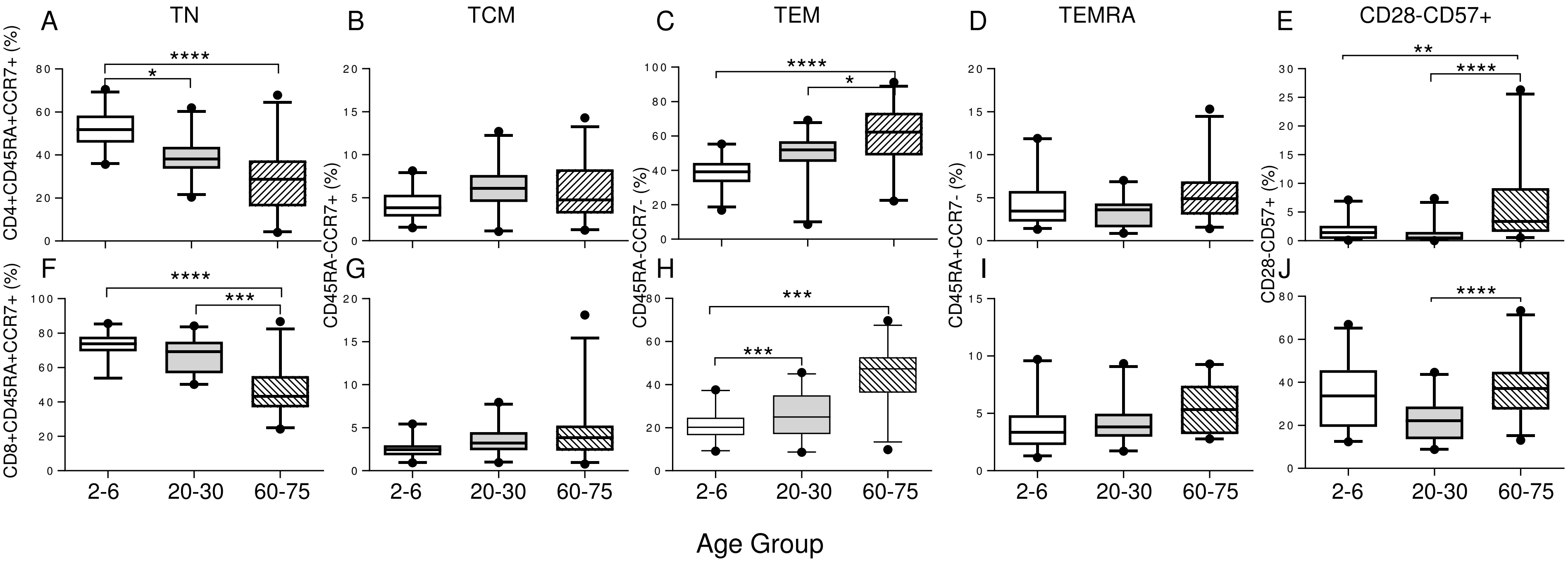

Supporting Fig.3. Variation in distribution of T cells subsets with age. Differentiation status was analysed for (A-E) $CD4^+$ T cells and (F-J) $CD8^+$ T cells. (A,F) $CD45RA^+CCR7^+$ naive T cells [TN]; (B,G) $CD45RA^-CCR7^+$ central memory T cells [TCM]; (C,H) $CD45RA^-CCR7^-$ effector T cells [TEM]; (D,I) $CD45RA^+CCR7^-$ terminally differentiated effector memory T cells [TEMRA] and (E,J) $CD28^-CD57^+$ terminally differentiated T cells. Data are shown for 68 subjects. In box and whisker plots, the horizontal bar indicates median frequency, the boxes extend 25th-75th percentile range and the whiskers indicate 95th percentiles. Statistical analysis was performed on samples using Kruskal-Wallis test, *$p<0.05$, **$p<0.01$, ***$p<0.001$, ****$p<0.0001$.

Supporting Fig.4.

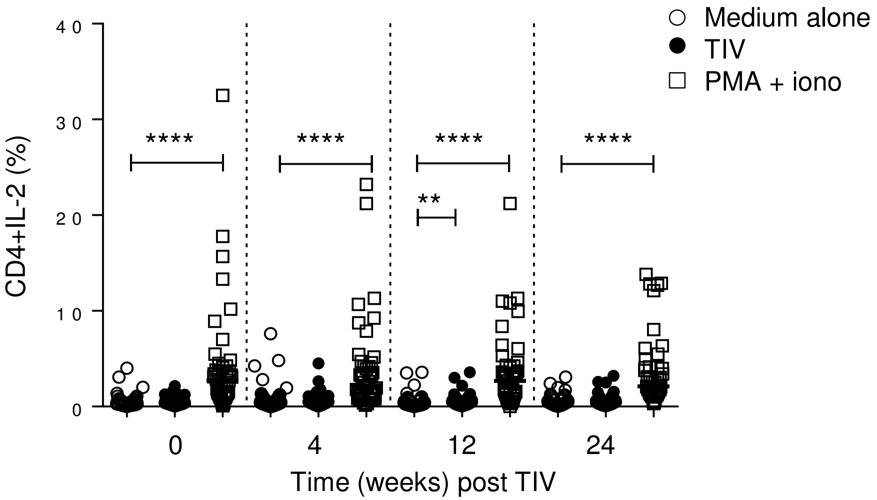

Supporting Fig.4. Limited induction of CD4 T cell IL-2 after TIV vaccination. CD4 IL-2 responses at baseline (Week 0) compared to 4, 12, and 24 weeks, post TIV vaccination. PBMC were cultured in medium alone (open round symbols) or in the presence of TIV antigens (close round symbols) or PMA/Ionomycin (open square symbols) for 5 hours. Data are shown from 64 subjects, each dot represents the frequency of IL-2^{+} CD4^{+} T cell in a single individual before and after vaccination, the horizontal line indicates the median frequency. Statistical analysis was performed on paired samples using Wilcoxon signed-rank test, **p<0.01, ****p<0.0001.

Supporting Fig.5.

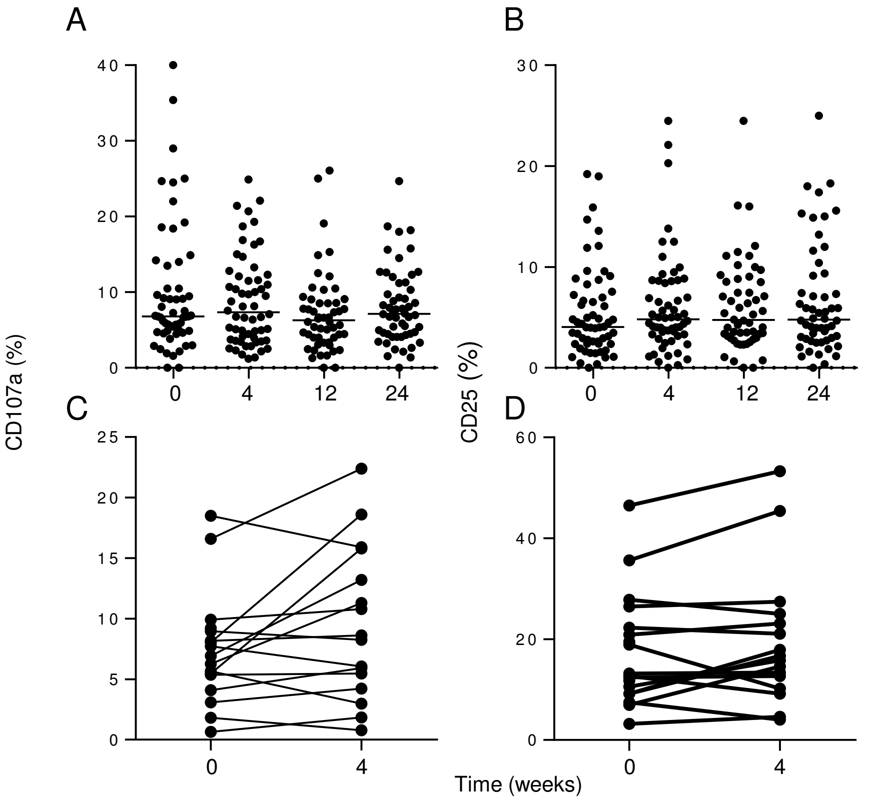

Supporting Fig.5. Limited effect of TIV vaccination on cytokine-induced NK cell degranulation and CD25 responses on NK cell subset distribution. (A, C) NK cell CD107a and (B, D) CD25 expression before and after vaccination, gated from CD56$^+$ cells. PBMC were cultured in high concentrations of cytokines alone (IL-12 5ng/ml + IL-18 50ng/ml). (A, B, E, F) Data are shown from 65 subjects receiving TIV and (C, D) 18 subjects receiving DTPiP. (E) Frequencies of CD56 and CD57-defined (CD56^{bright}, CD56^{dim}CD57$^-$ and CD56^{dim}CD57$^+$) NK cell subsets at baseline (Week 0) compared to 4, 12 and 24 weeks post TIV vaccination. (F) Frequency of NKG2C and CD57-defined NK cells subsets, (NKG2C$^-$CD57$^-$, NKG2C$^+$CD57$^-$, NKG2C$^-$CD57$^+$, and NKG2C$^+$CD57$^+$) at baseline (Week 0) compared to 4, 12 and 24 weeks post TIV vaccination, gated from CD56^{dim} cells. Statistical analysis was performed on paired samples using Wilcoxon signed-rank test.

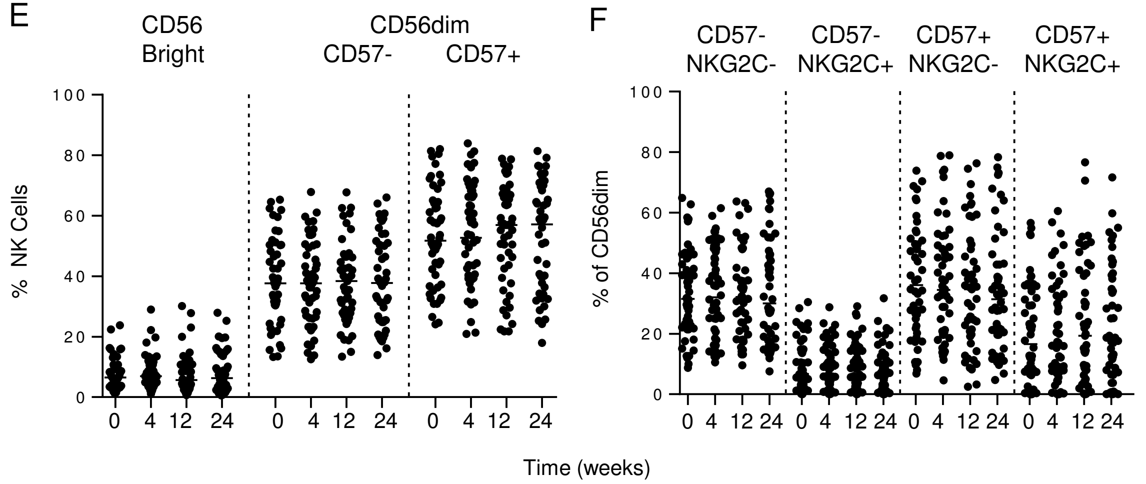

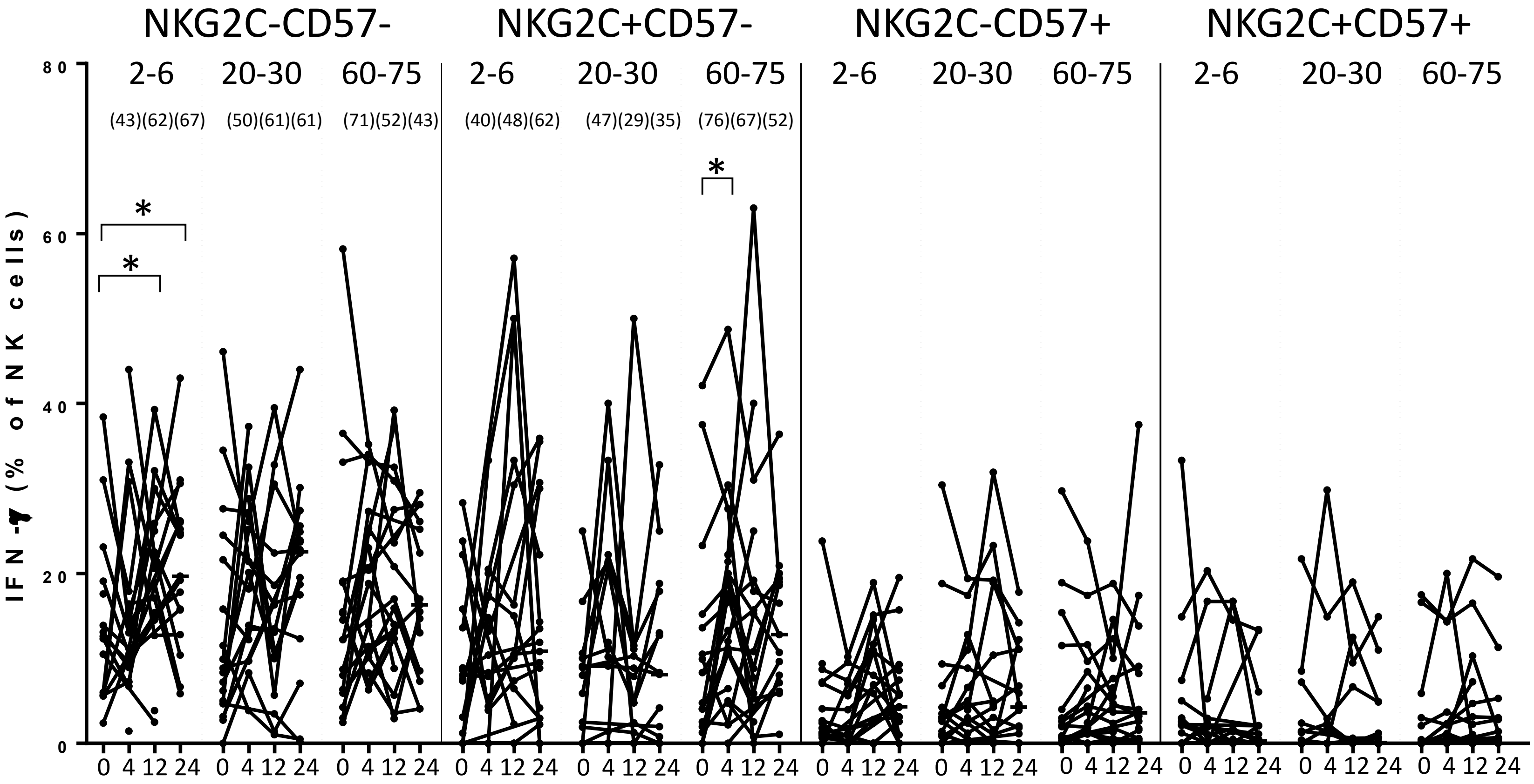

Supporting Fig.6. Frequency of NKG2C and CD57-defined NK cells subsets IFN-γ secretion split by age.

NKG2C$^-$CD57$^-$, NKG2C$^+$CD57$^-$, NKG2C$^-$CD57$^+$, and NKG2C$^+$CD57$^+$ subsets at baseline (Week 0) among the three age groups (2-6, 20-30, 60-75 years), compared to 4, 12 and 24 weeks post TIV vaccination, gated from CD56^{dim} cells. The percentage of responders for each age group are shown in brackets for each time point. Statistical analysis was performed on paired samples using Wilcoxon signed-rank test.
