# Supplementary material for: Enhancement of cytokine‐driven NK cell IFN‐γ production after vaccination of HCMV infected Africans
Source: Eur J Immunol. 2017 Apr 24;47(6):1040–50. doi: 10.1002/eji.201746974 (PMC5888140; doi:10.1002/eji.201746974)
Supplement: Supplementary file 2 — Peer review correspondence [file EJI-47-1040-s002.pdf]

European Journal of Immunology

Enhancement of cytokine-driven NK cell IFN-γ production after vaccination of HCMV infected Africans

Alansana Darboe, Ebrima Danso, Ed Clarke, Ama Umesi, Ebrima Touray, Rita Wegmuller, Sophie E. Moore, Eleanor M. Riley and Martin R. Goodier

Correspondence: Dr. Martin R. Goodier, Department of Immunology and Infection, London School of Hygiene and Tropical Medicine, London, UK

Review Timeline:	Submission date:	31-Jan-2017
	First Editorial decision:	16-Feb-2017
	First Revision received:	01-Mar-2017
	Accepted:	30-Mar-2017

Handling Executive Committee member: Prof. Annette Oxenius

Please note that the correspondence below does not include the standard editorial instructions regarding preparation and submission of revised manuscripts, only the scientific revisions requested and addressed.

First Editorial Decision - 16-Feb-2017

Dear Dr. GOODIER,

Manuscript ID eji.201746974 entitled "Enhancement of cytokine-driven NK cell IFN-Î³ production after vaccination of HCMV infected Africans." which you submitted to the European Journal of Immunology has been reviewed. The comments of the referees are included at the bottom of this letter.

A revised version of your manuscript that takes into account the comments of the referees will be reconsidered for publication. Should you disagree with any of the referees' concerns, you should address this in your point-by-point response and provide solid scientific reasons for why you will not make the requested changes.

You should also pay close attention to the editorial comments included below. **In particular, please edit your figure legends to follow Journal standards as outlined in the editorial comments. Failure to do this will result in delays in the re-review process.**

Please note that submitting a revision of your manuscript does not guarantee eventual acceptance, and

that your revision will be re-reviewed by the referees before a decision is rendered.

If the revision of the paper is expected to take more than three months, please inform the editorial office. Revisions taking longer than six months may be assessed by new referee(s) to ensure the relevance and timeliness of the data.

Once again, thank you for submitting your manuscript to European Journal of Immunology and we look forward to receiving your revision.

Yours sincerely,
Laura Soto Vazquez

On behalf of Prof. Annette Oxenius

Editorial Office
European Journal of Immunology
e-mail: ejied@wiley.com
www.eji-journal.eu
****
Reviewer: 1

Comments to the Author

The authors demonstrate that diphtheria, tetanus, pertussis and inactivated polio (DTPiP) and trivalent influenza vaccine (TIV) boosts IFN-gamma production ability in CD56brightCD57-NKG2C-, CD56dimCD57-NKG2C+ and â€“ NK cell populations in Gambians, which can be readily stimulated with IL-12 and IL-18 ex vivo. These include even elderly individuals, which have persistently carried HCMV presumably for prolonged periods of time. In contrast, individuals with an already quite responsive NK cell compartment, readily producing IFN-gamma prior to vaccination, do not significantly benefit in their NK cell reactivity from vaccinations, presumably because these cells are already considerably activated and readily produce IFN-gamma in response to cytokines. Presumably due to the high HCMV seroprevalence already at young age, no age-related benefit of vaccination on NK cell function was observed in the studied Gambians. From these data the authors conclude that in the elderly, NK cell responsiveness can be increased by vaccination, which might compensate for the lower responsiveness to cytokines in their NK cell compartment. This might be beneficial in NK cell sensitive disease settings.

The reported data are interesting, but important questions remain with respect to the relation between the

readily reactive CD57- NK cells in some donors and the CD56brightCD57-NKG2C-, CD56dimCD57-NKG2C+ and â€“ NK cells that seem to benefit from vaccination in others. This relationship should be analyzed in more detail, in order to facilitate the interpretation of the reported data.

Major comments:
1. Does elevated responsiveness correlate with natural influenza exposure? Is there any correlation to elevated influenza virus specific antibody titers in individuals with already high IFN-gamma production by NK cells after cytokine stimulation prior to vaccination?
2. Is the enhanced IFN-gamma production after vaccination comparable to age matched individuals from regions that are less exposed to HCMV and also other pathogens, like for example in the UK? Even so the authors have previously published on this topic in UK vaccines, they should comment on respective differences or even better compare them in the same assays side-by-side.
3. Are the readily IFN-gamma producing NK cells activated prior to vaccination and reflect rather the induced cytokines due to natural infections in the respective subgroup of vaccination insensitive individuals? Are NK cell activating cytokines, like IL-15 and IL-2, already elevated in the serum of individuals that do not benefit from vaccination for their NK cell responsiveness to cytokines? Are activation and proliferation markers like CD69, HLA-DR and Ki67 already elevated on NK cells in the respective individuals?
4. Along these lines, can the pre-vaccination serum of individuals that are not benefitting from vaccination, already induce NK cell reactivity in pre-vaccination PBMCs of individuals that then benefit from vaccination?
5. Do the authors ever observed CD56 and maybe even NKG2C up-regulation upon IL-15 or IL-2 stimulation of CD56dimCD57-NKG2C- or CD56dimCD57-NKG2C+ NK cell subpopulations?
6. Are any other NK cell produced cytokines, like TNF-alpha and GM-CSF, similarly affected by vaccination in elderly Gambians?
7. If the enhanced NK cell responsiveness, already prior to vaccination, does not reflect the influence of activation due to natural infections, it would be interesting to determine if the different NK cell subsets have just different thresholds of stimulating cytokines for IFN-gamma production. In the case of vaccination responders this threshold of CD56dimCD57-NKG2C+ and â€“ NK cells would be only overcome by a combined vaccine induced plus IL-12 and IL-18 triggered stimulation, while the ex vivo cytokines would be sufficient for the CD56+CD57-NKG2C- NK cell population.

In summary, the study investigates an interesting human population. However, pre-activation by natural infections or immune activation stimulating NK cell reactivity even without vaccination and thereby abolishing its benefit on NK cell activation needs to be investigated in more detail.

European Journal of Immunology

Reviewer: 2

Comments to the Author

In this paper the NK cell response to TIV influenza vaccine or to booster vaccination has been investigated in a Gambian population, aged 2-75. The good number of subjects recruited from this particular population, whose early and lifelong exposure to HCMV alters NK cell differentiation and influence NK cell abilities to respond to vaccines, represent the main strength of the study.

However the main conclusions drawn from this study are based on differences with previously analysed populations while the enhancement of cytokine-driven NK cell IFNg production highlighted in title consists in a modestly significant increase(that is clearly observed only in a part of the population and only at 6 months).

Major revisions

-In fig2, vaccination does not augment NK response to TIV when NK cells are costimulated with LCC, however, is TIV alone (w/o LCC) capable of increasing NK cell degranulation at any time point after vaccination? Is there any differential response among CD56dim subsets (57+/-, NKG2C+/-) in each individual examined?

-In fig3 baseline antibody levels (time 0) have been defined high(line 317), however baseline levels measured in a control population should be shown for comparison(e.g.Uk donors).

In panel D, 38 patients have been analyzed, however it is not clear whether they belong only to the 2-6 and 20-30 years groups or to all three groups.

NK cells from HCMV- and HCMV+ healthy donors should be added as controls in the experimental setting shown in panel D.

CD16 expression before and after vaccination should be examined at least for those subjects that have been analyzed for their ADCC ability.

-In fig4A, enhancement of IFN-g responses is statistically significant only in the 60-75 group (after TIV) at 24 weeks after vaccination, while it is only a tendency at the other time points. This should be clarified in the text.

A differential expression of IL12R and IL18R in the NK subsets from the various groups could explain the enhanced response to HCC?

-In fig.5 it appears that all subjects are reported together, not distinguished by age. It would be interesting to show results in the different age groups.

European Journal of Immunology

-In general it would be easier to appreciate NK cells features of the Gambian population if controls could be added in the various experimental settings and not only reported in the text.

Minor revisions

Line 57: CD161 expression is decreased in adaptive NK cells.

<u>First Revision – authors' response - 01-Mar-2017</u>

Reviewer: 1

Comments to the Author

The authors demonstrate that diphtheria, tetanus, pertussis and inactivated polio (DTPiP) and trivalent influenza vaccine (TIV) boosts IFN-gamma production ability in CD56brightCD57-NKG2C-, CD56dimCD57-NKG2C+ and – NK cell populations in Gambians, which can be readily stimulated with IL-12 and IL-18 ex vivo. These include even elderly individuals, which have persistently carried HCMV presumably for prolonged periods of time. In contrast, individuals with an already quite responsive NK cell compartment, readily producing IFN-gamma prior to vaccination, do not significantly benefit in their NK cell reactivity from vaccinations, presumably because these cells are already considerably activated and readily produce IFN-gamma in response to cytokines. Presumably due to the high HCMV seroprevalence already at young age, no age-related benefit of vaccination on NK cell function was observed in the studied Gambians. From these data the authors conclude that in the elderly, NK cell responsiveness can be increased by vaccination, which might compensate for the lower responsiveness to cytokines in their NK cell compartment. This might be beneficial in NK cell sensitive disease settings.

The reported data are interesting, but important questions remain with respect to the relation between the readily reactive CD57- NK cells in some donors and the CD56brightCD57-NKG2C-, CD56dimCD57-NKG2C+ and – NK cells that seem to benefit from vaccination in others. This relationship should be analyzed in more detail, in order to facilitate the interpretation of the reported data.

Major comments:

1. Does elevated responsiveness correlate with natural influenza exposure? Is there any correlation to elevated influenza virus specific antibody titers in individuals with already high IFN-gamma production by NK cells after cytokine stimulation prior to vaccination?

Response 1.

This is an interesting point as prior exposure could indeed potentially influence the in vivo impact of vaccination. We have compared the baseline antibody titres (shown in Figure 3) after splitting the cohort by those individuals making elevated IFN-[illegible] to cytokines after vaccination (responders) and those with no

enhancement (Split in the same way as shown in figure 4D,E). No difference in antibody titre was observed between responder and non-responder groups for TIV. (Responders median 657, IQR 500-806 and Non-responders median 625, 473-819).

This indicates that intrinsic properties of the NK cell compartment were determining the extent of cytokine responsiveness at baseline and that prior influenza exposures per-se do not influence whether individual responses to exogenous cytokines are elevated after vaccination (please see also the response to point 2, below).

2. Is the enhanced IFN-gamma production after vaccination comparable to age matched individuals from regions that are less exposed to HCMV and also other pathogens, like for example in the UK? Even so the authors have previously published on this topic in UK vaccines, they should comment on respective differences or even better compare them in the same assays side-by-side.

Response 2.

We have now included data on responses of UK donors to the same batch of TIV vaccine before vaccination and 1 month after vaccination, splitting the UK cohort into HCMV+ and HCMV negative individuals.

In-vitro responses to TIV are shown in Figure 2 (D-F). This data confirms that HCMV uninfected UK donors there is superior enhancement of antigen TIV antigen-driven IFN-γ responses after vaccination whereas HCMV+ UK subjects are similar to Gambians in their lack of enhancement of antigen responses. The results text is amended on lines 262-268 and the figure legend on lines 637-641.

In-vitro responses of UK individuals to cytokines in pre (baseline) and post-vaccination (4 weeks) samples are now also shown in Figure 4 (Figure 4B), alongside the data for the Gambian donors. Again, the UK cohort has been split into HCMV- and HCMV+ individuals to reveal more directly the influence of HCMV exposure. HCMV+ UK donors, who have weaker responses to cytokines at baseline have significant enhancement of cytokine-driven responses after vaccination, similar to the pattern observed in Gambian cytokine 'responder' individuals. Similar to the Gambian 'non-responders' Enhancement of cytokine-driven responses is not observed in HCMV- UK subjects, who already have strong responses to cytokines at baseline. The results section has been amended on lines 312-315, in the figure legend (lines 659-660, 667-668) and these points have been discussed (lines 391 to 394)

3. Are the readily IFN-gamma producing NK cells activated prior to vaccination and reflect rather the induced cytokines due to natural infections in the respective subgroup of vaccination insensitive individuals? Are NK cell activating cytokines, like IL-15 and IL-2, already elevated in the serum of individuals that do not benefit from vaccination for their NK cell responsiveness to cytokines? Are

activation and proliferation markers like CD69, HLA-DR and Ki67 already elevated on NK cells in the respective individuals?

Response 3.

We do not have any data on ex-vivo measurements of IL-2 and IL-15 in baseline serum samples. However, we have no analysed our ex-vivo data for functional markers, comparing vaccine insensitive individuals (with no enhancement of cytokine responses) to vaccine sensitive responder individuals (with elevated cytokine driven IFN-[illegible] responses after vaccination). Although we did not test ex-vivo frequencies of the early activation marker CD69 and the later marker HLA-DR in our study, we observed no difference in the ex-vivo frequency or MFI for CD25, the principal activation marker used in our study, marker between vaccine sensitive and insensitive individuals. We also observed no difference in the ex-vivo levels of CD107a% and IFN-[illegible] % between these groups. The values are given below for the reviewers' information.

(Median, IQR: CD25%- Responder= 1.19, 0.78-2.11 and Non-Responder 1.44, 1-2.24; Median, IQR: CD107a-Responder=2.31, 1.56-3.05 and Non-Responder=2.78, 2.1-3.88 and Median, IQR: IFN-[illegible] Responder=1.51, 0.67-2.24 and Non-Responder=1.16, 0.71-1.61), Background ex-vivo function for 60-75year olds: median, IQR Responder=2.31, 1.31-3.18 and Non-Responder 2.82, 1.82-4.32. This point has now been expanded in the discussion (lines 388-390).

Although we did not measure Ki67 expression, it is well known that this is mainly expressed in CD57- NK cell subsets, which tend to be elevated in frequency in non-responder individuals (Figure 4D,E). It is therefore possible that individual with already high responses at baseline have higher frequencies of cells with greater proliferative capacity. This point has been discussed further (lines 380-383)

4. Along these lines, can the pre-vaccination serum of individuals that are not benefitting from vaccination, already induce NK cell reactivity in pre-vaccination PBMCs of individuals that then benefit from vaccination?

Response 4.

We have now compared our in-vitro culture response data for pre-activation sera alone, without additional stimuli, between cytokine responder and non-responder groups. There was no difference between the Responder and Non-Responder groups in ex-vivo CD25, CD107a, IFN-[illegible] frequencies observed in medium with autologous baseline plasma. For the reviewers' information the background values in autologous plasma alone are given here: (CD25% Responders median, IQR =1.14, 0.67-1.98; and

European Journal of Immunology

non-responders median, IQR =1.12, 0.67-2.32;CD107a%, Responders: median, IQR= 3.44, 2.22-6.17 and non-responders, median, IQR = 4.50, 1.75-7.73; IFN- Responders, median, IQR= 0.4, 0-1.27 and non-responders median, IQR=0.22, 0.16-0.26)

This is consistent with there being no difference in potential NK cell activating cytokines between these groups and again indicates that a high capacity for cytokine response at baseline is likely to be an intrinsic property of NK cells or to be related to the distribution of cytokine responsive subsets as discussed above.

5. Do the authors ever observed CD56 and maybe even NKG2C up-regulation upon IL-15 or IL-2 stimulation of CD56dimCD57-NKG2C- or CD56dimCD57-NKG2C+ NK cell subpopulations?

Response 5.

We observed no increase in the frequency of NKG2C+ NK cells or of the MFI for this marker after cytokine or TIV stimulation in-vitro. In separate studies, we tend to observe an increase in CD56bright cells after long-term culture of NK cells with low concentrations of IL-15 (Goodier et al JI 2016). As we do not observe an overall increase in the frequency of CD56 bright cells in responder vs non-responder groups or after vaccination, this would again suggest that long-term in-vivo exposure to NK cell activating cytokines is not necessarily influence in-vitro cytokine responses in vaccine insensitive vs sensitive subjects.

6. Are any other NK cell produced cytokines, like TNF-alpha and GM-CSF, similarly affected by vaccination in elderly Gambians?

Response 6.

This is an interesting point, which would address whether the effect we are observing is IFN- specific or reflects a broader impact of vaccination on overall NK cell cytokine production. We have now raised this point in the discussion (line 407-409).

7. If the enhanced NK cell responsiveness, already prior to vaccination, does not reflect the influence of activation due to natural infections, it would be interesting to determine if the different NK cell subsets have just different thresholds of stimulating cytokines for IFN-gamma production. In the case of vaccination responders this threshold of CD56dimCD57-NKG2C+ and – NK cells would be only overcome by a combined vaccine induced plus IL-12 and IL-18 triggered stimulation, while the ex vivo cytokines would be sufficient for the CD56+CD57-NKG2C- NK cell population.

European Journal of
Immunology

Response 7.

We think it likely that the extent of exposure to agents which modify NK cell differentiation and function, in particular HCMV, will influence the threshold for activation within distinct differentiation subsets. We have addressed this point further in the discussion (line 395-400).

In summary, the study investigates an interesting human population. However, pre-activation by natural infections or immune activation stimulating NK cell reactivity even without vaccination and thereby abolishing its benefit on NK cell activation needs to be investigated in more detail.

Summary, response.

To address this point, we have now thoroughly analysed our data to assess whether there are differences in pre-activation both ex-vivo and in-vitro (medium alone cultures with pooled human AB+ plasma or autologous plasma) which could influence vaccine-induced enhancement of NK cell responses. No difference was observed in basal NK cell activation between vaccine responder and non-responder groups under any of these conditions, indicating that intrinsic differences in resting NK cells and in the distribution of resting NK cell subsets are more likely to be affecting subsequent vaccine induced responses. We have now included side-by side data for cytokine -induced responses in vaccinated UK subjects, which support the likelihood of distinct thresholds for NK cell activation according to the extent of previous exposure to NK cell modifying agents such as HCMV. More specifically, HCMV negative UK subjects already have high frequencies of IFN-� producing NK cells in response to cytokines at baseline, which are not further enhanced by vaccination, whereas there is a recovery of cytokine induced IFN-� in CMV+ subjects.

Reviewer: 2

Comments to the Author

In this paper the NK cell response to TIV influenza vaccine or to booster vaccination has been investigated in a Gambian population, aged 2-75. The good number of subjects recruited from this particular population, whose early and lifelong exposure to HCMV alters NK cell differentiation and influence NK cell abilities to respond to vaccines, represent the main strength of the study.

However the main conclusions drawn from this study are based on differences with previously analysed populations while the enhancement of cytokine-driven NK cell IFNg production highlighted in title consists in a modestly significant increase(that is clearly observed only in a part of the population and only at 6 months).

Response: We observed enhanced cytokine-driven NK cell responses at all time points between 4, 12 weeks and 6 months after vaccination. As indicated in the materials and methods. The data presented in figure 4a were analysed with an Anova test, after correction for multiple comparisons. However, for reasons of clarity and consistency with the tests used throughout, we have now used a paired non-parametric tests (Wilcoxon-signed rank) comparing the baseline response separately with the IFN-? responses observed at each post vaccination time point figure 4A.

Major revisions

1.-In fig2, vaccination does not augment NK response to TIV when NK cells are costimulated with LCC, however, is TIV alone (w/o LCC) capable of increasing NK cell degranulation at any time point after vaccination? Is there any differential response among CD56dim subsets (57+/-, NKG2C+/-) in each individual examined?

Response 1.

Similar to the data obtained in the presence of low concentrations of cytokines (LCC), we observed no post vaccination enhancement of NK cell responses to vaccine antigen alone. We do not observe enhanced antigen-driven responses with any of the CD57/NKG2C defined NK cell subsets for any of the individuals studied. These points have been mentioned in the text (results, lines 262-264).

2. -In fig3 baseline antibody levels (time 0) have been defined high (line 317), however baseline levels measured in a control population should be shown for comparison (e.g.Uk donors).

Response 2.

We have now stated the baseline UK antibody levels against TIV in 17 UK donors both before and after vaccination (Results lines 291 - 292). We have also given the data for the three age groups within the Gambian cohort for comparison (lines 285-289).These data were obtained using the same antigen and standardised ELISA protocols. This comparison demonstrates that, despite likely geographical variations in virus strains and in the extent of seasonal exposure, similar levels of antibody were observed in UK and in Gambian children and 20-30 year olds both before and after vaccination.

3. In panel D, 38 patients have been analyzed, however it is not clear whether they belong only to the 2-6 and 20-30 years groups or to all three groups.

Response 3.

This experiment was performed on cells remaining after the main series of experiments, because of limited blood volumes collected from children (3 donors shown), the donors are mainly 20-30 (17 donors shown) and 60-75 year old individuals (18 donors shown). The number of individuals from each age-defined group is now stated in the legend for figure 3 (lines 654-655).

4. NK cells from HCMV- and HCMV+ healthy donors should be added as controls in the experimental setting shown in panel D.

Response 4.

We have now included data for HCMV+ and HCMV - UK individuals as a new sub-figure (3E).

5. CD16 expression before and after vaccination should be examined at least for those subjects that have been analyzed for their ADCC ability.

Response 5.

We have analysed our data on the effect of pre and post vaccination plasma antibody on CD16 expression. As expected, and conversely to the enhancement of CD107a, TIV stimulation in the presence of both pre and post vaccination plasma resulted in CD16 down regulation in post-vaccination NK cells: (Medium alone: Pre vaccination CD16 MFI, median, IQR = 6539, 5137-7434; post vaccination, median IQR 5673, 5270-8823; TIV: Pre vaccination CD16 MFI, median, IQR = 1107,810-1642; post vaccination, median IQR 713, 562-1037).

This data has now been included in the text (lines 298-302).

6. -In fig4A, enhancement of IFN-g responses is statistically significant only in the 60-75 group (after TIV) at 24 weeks after vaccination, while it is only a tendency at the other time points. This should be clarified in the text.

Response 6.

The data shown have been analysed by trend Anova which indicates that the difference from baseline is significant across all of the time points (see also our initial response for the reviewer above). However, for clarity we have now performed paired non-parametric tests comparing baseline values with each post-vaccination timepoint and amended figure 4a accordingly.

7. A differential expression of IL12R and IL18R in the NK subsets from the various groups could explain the enhanced response to HCC?

Response 7.

Although we have not tested the ex-vivo expression of these cytokine receptors, their potential affect cytokine responses both before and after vaccination is an important point. We have discussed this point in the context of what is known about the distribution of these receptors at different stages of NK cell differentiation according to CD57 expression (lines 395-400).

8. -In fig.5 it appears that all subjects are reported together, not distinguished by age. It would be interesting to show results in the different age groups.

Response 8.

We have now reanalysed this data according to the different age groups and put this as a supplementary figure (Figure S6). Overall heightened responses to cytokines were observed after vaccination within the CD57-NKG2C- and CD57-NKG2C- subsets for all three age groups. This data has been mentioned in the results on lines 343-345.

9. -In general it would be easier to appreciate NK cells features of the Gambian population if controls could be added in the various experimental settings and not only reported in the text.

Response 9.

We have now included the data for baseline and post-vaccination responses (4 weeks) for UK donors. In-vitro re stimulations with TIV antigen are now shown alongside data from Gambian subjects in figure 2D,E,F, antibody dependent responses from UK subjects are shown in Figure 3E and cytokine driven responses are shown in figure 4B. Please also refer to our response to reviewer 1, point 2.

Minor revisions

10. Line 57: CD161 expression is decreased in adaptive NK cells.

We have altered this text at lines 57-58.

Second Editorial Decision - 13-Mar-2017

Dear Dr. GOODIER,

It is a pleasure to provisionally accept your manuscript entitled "Enhancement of cytokine-driven NK cell IFN-Î³ production after vaccination of HCMV infected Africans." for publication in the European Journal of Immunology. For final acceptance, please follow the instructions below and return the requested items as soon as possible as we cannot process your manuscript further until all items listed below are dealt with.

Please note that EJI articles are now published online a few days after final acceptance (see Accepted Articles: http://onlinelibrary.wiley.com/journal/10.1002/(ISSN)1521-4141/accepted). The files used for the Accepted Articles are the final files and information supplied by you in Manuscript Central. You should therefore check that all the information (including author names) is correct as changes will NOT be permitted until the proofs stage.

We look forward to hearing from you and thank you for submitting your manuscript to the European Journal of Immunology.

Yours sincerely,
Laura Soto Vazquez

on behalf of Prof. Annette Oxenius

Editorial Office
European Journal of Immunology
e-mail: ejied@wiley.com
www.eji-journal.eu
